# Fungal Communities of the Pine Wilt Disease Complex: Studying the Interaction of Ophiostomatales With *Bursaphelenchus xylophilus*

**DOI:** 10.3389/fpls.2022.908308

**Published:** 2022-06-14

**Authors:** Cláudia S. L. Vicente, Miguel Soares, Jorge M. S. Faria, Margarida Espada, Manuel Mota, Filomena Nóbrega, Ana P. Ramos, Maria L. Inácio

**Affiliations:** ^1^MED - Mediterranean Institute for Agriculture, Environment and Development & CHANGE - Global Change and Sustainability Institute, Institute for Advanced Studies and Research, Universidade de Évora - Pólo da Mitra, Évora, Portugal; ^2^Instituto Nacional de Investigação Agrária e Veterinária (INIAV, I.P.), Quinta do Marquês, Oeiras, Portugal; ^3^Laboratório de Patologia Vegetal “Veríssimo de Almeida” (LPVVA), Instituto Superior de Agronomia (ISA), University of Lisbon, Lisboa, Portugal; ^4^MED - Mediterranean Institute for Agriculture, Environment and Development & CHANGE - Global Change and Sustainability Institute, Department of Biology, Universidade de Évora - Pólo da Mitra, Évora, Portugal; ^5^GREEN-IT Bioresources for Sustainability, ITQB NOVA, Oeiras, Portugal

**Keywords:** biocontrol, blue-stain fungi, diversity, ecological interactions, mycoflora, pinewood nematode

## Abstract

Considered one of the most devastating plant–parasitic nematodes worldwide, *Bursaphelenchus xylophilus* (commonly known as pinewood nematode, PWN) is the causal agent of the pine wilt disease in the Eurasian coniferous forests. This migratory parasitic nematode is carried by an insect vector (*Monochamus* spp.) into the host tree (*Pinus* species), where it can feed on parenchymal cells and reproduce massively, resulting in the tree wilting. In declining trees, PWN populations are strongly dependent on fungal communities colonizing the host (predominantly ophiostomatoid fungi known to cause sapwood blue-staining, the blue-stain fungi), which not only influence their development and life cycle but also the number of individuals carried by the insect vector into a new host. Our main aim is to understand if PWN-associated mycobiota plays a key role in the development of PWD, in interaction with the PWN and the insect vector, and to what extent it can be targeted to disrupt the disease cycle. For this purpose, we characterized the fungal communities of *Pinus pinaster* trees infected and non-infected with PWN in three collection sites in Continental Portugal with different PWD temporal incidences. Our results showed that non-infected *P. pinaster* mycoflora is more diverse (in terms of abundance and fungal richness) than PWN-infected pine trees in the most recent PWD foci, as opposed to the fungal communities of long-term PWD history sites. Then, due to their ecological importance for PWN survival, representatives of the main ophiostomatoid fungi isolated (*Ophiostoma, Leptographium*, and *Graphilbum*) were characterized for their adaptative response to temperature, competition in-between taxa, and as food source for PWN. Under the conditions studied, *Leptographium* isolates showed promising results for PWN control. They could outcompete the other species, especially *O. ips*, and significantly reduce the development of PWN populations when compared to *Botrytis cinerea* (routinely used for PWN lab culturing), suggesting this to be a natural antagonist not only for the other blue-stain species but also for the PWN.

## Introduction

Pine wilt disease (PWD) is a devastating disease that affects mainly coniferous trees of the *Pinus* genus. The plant parasitic nematode *Bursaphelenchus xylophilus* (Steiner & Buhrer) Nickle, commonly known as the pinewood nematode (PWN), is responsible for the onset of PWD symptoms, namely, the characteristic needle yellowing and wilting as a result of shoot desiccation and chlorosis, that can lead to tree death in under 2 months (Futai, 2013). The PWN was first identified in North America, where it is believed to be an endemic. In its native range, the PWN only seldom causes host disease, generally in weakened native species or on exotic introduced pine species (Dwinell, 1997). There, the disease has a low expression because of the co-evolution of the PWN with native pines. In the beginning of the 20th century, in the Japanese islands, extensive ranges of pine forest began developing PWD symptoms, and the PWN was identified soon after (Mamiya, 1983). Since then, PWD has spread throughout the Asian continent causing strong ecological and economic impacts in Japan, China, Korea, and Taiwan (Vicente et al., 2012). In 1999, Portugal became its entry point into the European continent leading to the enforcement of strong containment strategies by national authorities and the EU and was set under quarantine (Mota et al., 1999). However, by 2008, the Portuguese continental area and Madeira Island (Fonseca et al., 2012) were considered affected and even more restringing measures were applied (Rodrigues et al., 2015). Several outbreaks have been reported in border Spanish forests (Abelleira et al., 2011). The PWN is now considered an A2 quarantine pest in the EU and a strong yearly investment is made in its containment and eradication for fear of a European pandemic in its susceptible pine forests (Robertson et al., 2011; Fonseca et al., 2012; de la Fuente and Saura, 2021).

The development of PWD and the intensity of its symptomatology are highly dependent on environmental and biological factors (Liu et al., 2021). Besides its causal agent, the PWN, other organisms are known to influence the development of PWD. Fungi are of great importance for the PWN. For dispersal, the PWN depends on an insect vector, the *Monochamus* genus, that carries and transmits the parasite to the new susceptible healthy host pines. Once inside the new host, the PWN enters the resin canals, attacks the epithelial cells, and causes extensive damage as it moves through the canal system and rapidly reproduces. During this reproductive phase on dead or decaying wood, the PWN depends necessarily on local fungal flora as a source of food for rapid multiplication (Futai, 2013). A weakened pine tree is more easily attacked by the fungi transmitted by bark beetles (Coleoptera: Scolytidae) that further increase the symptomatology of wilting and function as a necessary food source for the PWN. Thus, these fungi are essential for nematode survival and reproduction (Vicente et al., 2021). Generally called blue-stain fungi, referring to the damage they cause, namely blue or even black discoloration of the sapwood of trees, mostly on conifers (Harrington, 1993; Kim et al., 2003; Zhou et al., 2006; Six, 2012), these bark beetle vectored fungi are also known as “ophiostomatoid fungi” (Kirisits, 2007). Within this group, Ophiostomatales, comprising six genera, *Ophiostoma* sensu stricto (*s. str*.), *Raffaelea s. str., Ceratocystiopsis, Fragospaeria, Graphilbum*, and *Leptographium senso lato* (s. l.) are clearly separated from fungi of the Microascales order, namely *Ceratocystis* sp. (de Beer et al., 2013). *Ceratocystis, Ceratocystiopsis*, and *Ophiostoma* fungi and their related anamorphs could be distinguished based on the chemical composition of their cell walls. Besides chitin, cell walls of *Ophiostoma* also contain cellulose and rhamnose, which is quite unusual for ascomycete fungi. In contrast, the cell walls of *Ceratocystis* and *Ceratocystiopsis* spp. consist mainly of chitin. The cell walls of *Ophiostoma* fungi make them highly tolerant to cycloheximide that inhibits the protein synthesis of most eukaryotic organisms (Harrington, 1981), while species of *Ceratocystis* and *Ceratocystiopsis* are very sensitive to even low concentrations of this antibiotic (Kirisits et al., 2002; Plattner et al., 2009; Reid et al., 2010).

The hypothesis of PWN nutritional dependency in relation to some species of ophiostomatoid fungi inhabiting pine trees is supported by significant observations: (1) PWN propagates at higher rates in fungus-inoculated wood than in uninoculated wood (Maehara and Futai, 2000); and the number of PWN individuals transferred to a beetle is much higher in fungus-colonized wood than in fungal-free wood (Maehara and Futai, 1996). Maehara and Futai (1996, 1997) showed that *O. minus*-inoculated wood-blocks of *P. densiflora* presented a higher percentage of PWN transferred by beetles than when inoculated with *Trichoderma* spp. or in uninoculated blocks. Beetles emerging from pupal chambers having an intense colonization by blue-stain fungi vectored higher numbers of dauer juveniles (J_IV_), highlighting the relation between the PWN population and the fungal species present in the infected tree (Maehara and Futai, 2005). These results and further work (to Maehara et al., 2006; Maehara, 2008; Niu et al., 2012; Futai, 2013; Pimentel et al., 2021) undoubtedly suggest a mutualistic symbiosis between the nematode and certain fungi, possibly because of a long-term co-evolution. This effect is not surprising considering the mycetophagous status of *Bursaphelenchus* nematodes and might represent the trace of an evolutionary transition from this fungal-feeding behavior to the plant parasitic activity of *B. xylophilus* (Kanzaki et al., 2014).

Although there is strong evidence of fungal involvement in the expression of PWD, this subject is controversial, and to understand the complex dynamics of PWD it is crucial to identify the fungal community in declining trees, ascertaining which species have a role in PWN feeding, reproduction, and dispersal. The comprehensive scenario of the fungal community in the PWN life cycle, through a spatio-temporal analysis of the mycobiota associated with the nematode and the susceptible host tree, constitutes a pioneering approach in Portuguese maritime pine stands with a high prevalence of PWD. The present study characterizes the: (i) fungal communities in non-infected and PWN-infected *P. pinaster* trees from three contrasting PWD focus on Continental Portugal and (ii) adaptative responses of ophiostomatoid fungi (genus *Ophiostoma, Leptographium* and *Graphilbum*) inhabiting Portuguese pine stands to temperature, antagonism, and PWN interaction due to their well-recognized history with PWN.

## Materials and Methods

### Wood Sampling and Processing, and Nematode Collection

Three surveys were conducted in mainland Portugal during the period of October 2019 in Seia (Guarda District, Central Portugal) in January 2020 in Companhia das Lezírias (Santarém, Central Portugal) and June 2020 in Tróia (Setúbal District, Coast of Portugal) (Table 1). The first location (Seia) is considered a transitional PWD focus, where the disease was identified in 2018–2019. The other two locations, Companhia das Lezírias and Tróia, are known as long-term PWD foci, nearby the first PWN detection site in Portugal (Mota et al., 1999). At each site, 10 PWD-symptomatic trees and 10 non-symptomatic trees were randomly selected according to the wilting/defoliation classification: 0, no symptoms; 1, first branch with yellow needles; 2, yellow tree canopy; 3, yellow to reddish tree canopy; and 4, brown canopy to dead tree (Cadahia et al., 1991). Wood disk were collected and stored in plastic bags for transportation to INIAV I.P. laboratories (Oeiras, Portugal). Concerning wood processing, cutting was conducted thoroughly with disinfection of the mechanical woodworking band saw with 70% (v/v) ethanol in-between cuttings and beginning with non-symptomatic trees. Approximately 100 g of wood pieces were used for nematode extraction by the modified tray method (Whitehead and Hemming, 1965). The rest of the material was stored at 4°C. After 48 h of incubation, samples were observed under a stereoscopic microscope (Olympus SZX12, Tokyo, Japan) to detect PWN. The following classes were considered to score nematode abundance in 100 g of wood: class 0, no nematodes recovered; class I, <50 nematodes; class II, between 51 and 200 nematodes; class III, between 201 and 1,000 nematodes; class IV, with 1,001 to 5,000 nematodes; and class V, more than 5,000 nematodes. The suspension of PWN was collected and stored in sterile 50 mL Falcon tubes. All material (wood pieces, and nematode suspensions) were stored at 4°C until further processing. The selection of *P. pinaster* trees for fungal isolation was based on: PWD symptomology and presence of PWN in higher proportion to other tree-inhabiting nematodes.

**Table 1:** Characterization of geographic and climate conditions for each collection site (Seia, Tróia, Companhia das Lezírias).

**Collection Sites**					***Pinus pinaster*** **samples**			
**Location**	**GPS**	**Tmax (**°**C)^**a**^**	**Tmin (**°**C)^**a**^**	**Precipitation (mm)^**a**^**	**PWD symptoms**	**Tree ID**	**PWD symptoms^**b**^**	**PWN class^**c**^**
Seia (S)	40°15'57.0”N	18	0.5	50–100	No	S1	0	0
	7°42'47.6”W					S2	0	0
						S13	0	0
						S19	0	0
						S32	0	0
					Yes	S35	3	IV
						S36	3	IV
						S38	3	IV
						S39	3	III
						S40	3	III
Companhia das Lezírias (L)	38°49'17.6“N	10	1.5	50–100	No	L7	0	0
	8°52'20.5”W					L8	0	0
						L9	0	0
						L10	0	0
						L11	0	0
					Yes	L2	4	III
						L3	3	III
						L4	3	IV
						L5	3	III
						L6	4	IV
Tróia (T)	38°28'07”N	20	0.5	1–5	No	T8	0	0
	8°52'18”W					T9	0	0
						T10	0	0
						T11	0	0
						T12	0	0
					Yes	T1	4	IV
						T2	4	III
						T3	4	II
						T4	4	III
						T7	4	IV

*Classification of Pinus pinaster trees sampled (disease symptoms and nematode class)*.

*^a^Average values at collection time obtained from IPMA Bulletins (Portugal) (Instituto Português do Mar e da Atmosfera (IPMA), 2019, 2020); ^b^Symptoms Class: 0, no symptoms; 1, one yellow branch; 2, yellow canopy; 3, yellowish to reddish canopy; 4, brown canopy—dead tree. ^c^Nematode Class (per 100 grams of wood): 0, no nematodes; I, < 50 nematodes; II, 51–200 nematodes; III, 201–1000 nematodes; IV, 1001–5000 nematodes; V, > 5001 nematodes*.

### Isolation of Ophiostomatales and Other Wood-Inhabiting Fungi

Under aseptic conditions, wood pieces from the selected trees were firstly cut into small fragments, surface sterilized with 70% ethanol (v/v) for 15 s, followed by serial washes with sterile distilled water, and dried in a sterilized filter paper. Following, surface-sterilized fragments were transferred to 2% (w/v) malt extract agar (MEA, Difco) supplemented with 200 mg/l of cycloheximide and 100 mg/l streptomycin. Plates were incubated at 25°C in the dark and monitored daily for fungal growth for at least 1 week. Hyphal tips of emerging colonies were transferred to fresh MEA plates. Pure cultures of the fungal isolates were grouped according to culture morphology. Representatives from each group were selected for further identification and characterization and deposited in the culture collection of INIAV institute (Micoteca da Estação Agronómica Nacional (MEAN).

### DNA Extraction, Amplification, and Phylogenetic Analysis

For each fungal representative, fresh mycelium was grown in 2% MEA and incubated for 7 days at 25°C. Total DNA was extracted using the DNeasy Plant mini kit (Qiagen GmbH, Hilden, Germany) following the protocol provided by the manufacturer. Partial gene sequences were determined for the internal transcribed spacer region (ITS), β-tubulin (TUB), elongation factor 1-α (TEF), and calmodulin (CAL) using primers listed in Supplementary Table 1 (White et al., 1990; Gardes and Brunts, 1993; Glass and Donaldson, 1995; Carbone and Kohn, 1999; Duong et al., 2012; Marincowitz et al., 2015). For a final volume of 25 μl, the PCR reaction mixture consisted of 2.5 μl of 10x reaction Buffer, 0.5U Supreme NZYTaq II (NZYTECH, Portugal), 1.0 μl dNTP mix (10 mM), 1.25 μl of each primer (10 uM), 1.25 μl Mg2+ (50 mM), and 2 μl template DNA. The PCR conditions were: (i) initial denaturation at 95°C for 5 min; (ii) 34 cycles of denaturation at 94°C for 30 s, annealing at 48–52°C (depending on the marker gene), and extension at 72°C for 15–30 s; and (iii) final extension at 72°C for 10 min. PCR products were cleaned using EXO-SAP (Exonuclease I and FastAP™ Thermosensitive Alkaline Phosphatase, ThermoScientific, CA, USA) following the manufacturer's recommendations. The amplified PCR products were sequenced at Stabvida (Costa da Caparica, Portugal) using the same primers used for their amplification. All sequences from selected fungal isolates were deposited in NCBI GenBank.

Sequences were used as the query at NCBI GenBank for preliminary identification using BLAST tool (https://blast.ncbi.nlm.nih.gov/). Published sequences from closely related species were retrieved from the database (Supplementary Table 2). Different datasets of ITS, TUB, and CAL gene sequences were compiled based on the species complex or genera. Multiple sequence alignment was conducted using MAFFT v7.0 (Katoh and Standley, 2013) and trimmed with trimAl v.1.4.1 (Capella-Gutiérrez et al., 2009). Phylogenetic trees were reconstructed for each dataset using Maximum likelihood (ML) method, and the best fitting substitution model selected using the Akaike Information Criterion (AIC) estimated in the Model Testing plugin from CLC Main Workbench 21.0.5 software (QIAGEN, Aarthus A/S). Final adjustments were made in iTOL (https://itol.embl.de) (Letunic and Bork, 2021). Phylogenetic trees were supported by 1,000 bootstrap to assess node support and overall robustness of the tree topology.

### Growth Studies and Direct Inhibition Antagonistic Trials

Representatives of the ophiostomatoid isolates were selected for growth studies and antagonistic trials. For the growth studies, mycelium of each fungal representative was cultured on three 9-cm (Ø) Petri dishes containing 2% MEA (Difco®). Plates were inoculated with a 7 mm diameter mycelial plug taken from the margin of 5 days actively growing cultures and incubated in the dark at seven different temperatures: 5, 10, 20, 25, 30, 35, and 40°C. The radial growth of each plate was measured every 24 h, until the colonies reached 7 cm in diameter.

To assess the direct inhibition antagonism between ophiostomatoid-like isolates, different combinations were tested by dual culture assay (Royse and Ries, 1977; Almeida et al., 2020). A mycelium plug from pure cultures of the selected fungal isolates were placed on opposite sides of a 9-cm potato dextrose agar (PDA, Difco) plate, ~1 cm to the edge of the plate. Each combination was repeated three times and incubated at 25°C in dark conditions for 5 days. Measurements of the radial growth (from the edge of the plug until the end of the colony) were taken daily. The inhibition percentage was calculated by I (%) = [(R1-R2)/R1]x100, where R1 was the radial growth of the potential antagonist and R2 was the radial growth of the test organism.

### PWN Feeding Trials in the Different Ophiostomatoid Fungi

The reference culture *B. xylophilus* BX013.003 (N 39°43'338”, W 9°01'557”) from INIAV Nematology collection (INIAV I.P., Oeiras) was used in this study. Nematodes were routinely cultured in the non-sporulating strain *Botrytis cinerea* grown on autoclaved barley seeds at 25°C. Prior to the experiments, the nematodes were extracted using the modified Baermann funnel technique (Whitehead and Hemming, 1965) and counted under an Olympus SZX12 (Tokyo, Japan) stereomicroscope for concentration adjustment.

The selected ophiostomatoid isolates were tested on feeding trials with PWN. Cultures of *B. cinerea* were used as a positive control for PWN population growth. For each isolate and control tested, six PDA plates were previously prepared and grown for 7 days at 25°C. Freshly extracted nematodes, mixed life-stages, were adjusted for a concentration of 1,000 nematodes per ml of distilled water, and 500 μl (ca. 500 nematodes) was inoculated into PDA plates containing the chosen isolates. Seven days after PWN inoculation, the nematodes were extracted by the modified Baermann method for 24 h and counted under an Olympus SZX12 stereomicroscope. The approximate total number of PWN was calculated for the total volume of the nematode suspension (final volume adjusted to 20 mL). Six replicates were conducted for each isolate. Each counting was repeated three times to correctly access the number of nematodes of each sample according to Aikawa and Kikuchi (2007).

### Data Analysis

The relative frequency of isolation (%) was estimated as the number of isolates of each order divided by the total number of species isolated and multiplied by 100 (Chang et al., 2017). To characterize the species diversity and richness of fungal communities from the different sites, the following indexes were calculated in PAST 4.02 software (Hammer et al., 2001): Simpson diversity index (S1-D), Shannon diversity index (H), dominance (D), evenness (E), and Sørensen index. Statistical analyses were performed using R in jamovi software 1.6.23 (R Core Team, 2020; The jamovi project, 2021). Kruskal–Wallis test (non-parametric one-way analysis of variance, ANOVA) was used to infer differences in the total number of PWN grown in the different ophiostomatoid isolates; the data was also analyzed using DSCF pairwise comparison (*p* < 0.05) for multiple mean comparisons.

## Results

### Fungal Isolation and Diversity Analysis

Three surveys were conducted before the maturation feeding phase of the insect vector, when the insect vector is not yet flying (and carrying PWN) for new host trees. The three locations were sorted by long-term PWD foci (Tróia; and Companhia das Lezírias) and transitional or recent PWD focus (Seia), aiming the description of shifts in the fungal communities as the result of disease history in these locations. In each survey (Table 1), a total of 10 *P. pinaster* trees were considered for fungal isolation, namely, 5 non-symptomatic trees with symptoms and nematode class 0; and 5 PWD symptomatic trees with symptoms between class 3 and 4, and nematode concentration ranging from class III (201–1,000 nematodes per 100 g of wood) and class IV (1001-5000 nematodes per 100 g of wood). The presence of PWN was only detected and identified in symptomatic *P. pinaster* trees. Hereafter, symptomatic trees will be designated as PWN-infected, and non-symptomatic trees will be referred to as non-infected.

**Figure 1:**
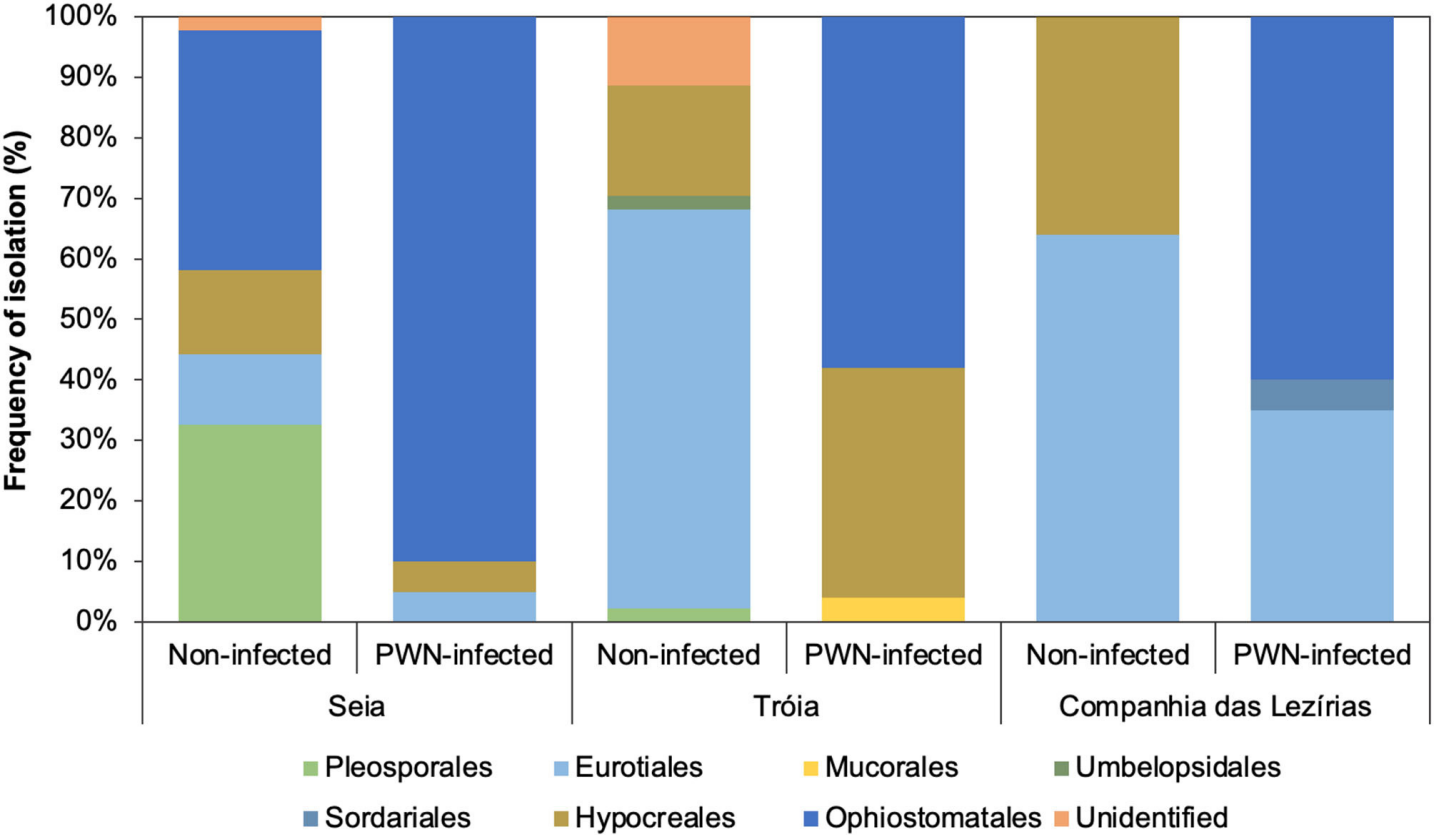
Frequency of isolation (%) of the main Ophiostomatales orders isolated in this study.

In terms of the total number of fungal isolations, 242 isolates were obtained from *P. pinaster* trees (45 from Companhia das Lezírias; 94 from Tróia; and 103 from Seia). Culture morphology and color supported by molecular marker sequencing were used for the taxonomic identification of each fungal isolate. Overall, the most abundant orders were Ophiostomatales (58% in Tróia, 60% in Companhia das Lezírias, and 90% in Seia) mostly detected in PWN-infected *P. pinaster*, followed by orders Pleosporales (2% in Companhia das Lezírias and 33% in Seia) and Eurotiales (64% in Companhia das Lezírias and 66% in Tróia) only in non-infected trees (Figure 1). The representative genera of Ophiostomatales were *Ophiostoma* with 34% (17 isolates in non-infected trees in Seia; and 65 isolates in all PWN-infected trees), *Leptographium* with 10% (24 isolates only in PWN-infected *P. pinaster*), *Graphilbum* with 2.1% (5 isolates in PWN-infected *P. pinaster*), and *Sporothrix* with 0.1% (one isolate detected in PWN-infected trees). In the orders Pleosporales and Eurotiales, the most predominant genera were *Alternaria* with 6.2% (15 isolates in non-infected trees) and *Penicillium* with 23.1% (56 isolates in non-infected trees). Only 2.5% of the fungal isolates were not identified as being classified as unidentified. Based on this general identification, and since the number of isolates was uneven between collection sites (Supplementary Figure 1), diversity indexes (Simpson 1-D and Shannon-H) varied widely among locations (Table 2). The fungal communities in the non-infected *P. pinaster* were more diverse (S1-D = 0.71; SH = 1.43) than in PWN-infected *P. pinaster* (S1-D = 0.46; SH = 0.87) in Seia. However, for the case of Companhia das Lezírias and Tróia, the results were different being PWN-infected more diverse in what concerns fungal composition than non-infected *P. pinaster*. For each site, similarity Sørensen index indicated that fungal communities from non-infected and PWN-infected trees were dissimilar, this observation being clearer in Tróia (S = 0.182) and Companhia das Lezírias (S = 0.2) than in Seia (S = 0.6). The PCoA analysis clearly separated PWN-infected fungal communities from non-infected *P. pinaster* fungal communities in Seia and Tróia (Supplementary Figure 2). Fungal communities from both PWN-infected and non-infected *P. pinaster* in Companhias das Lezirias clustered together, denoting no separation.

**Table 2:** Ecological diversity indexes (taxa = order) of fungal communities from non-infected and PWN-infected *Pinus pinaster* trees sampled.

**Diversity indexes**	**Seia**		**Companhia das Lez**í**rias**		**Tróia**	
	**Non-infected**	**PWN-infected**	**Non-infected**	**PWN-infected**	**Non-infected**	**PWN-infected**
Taxa S	6	4	4	6	6	5
Individuals	43	60	25	20	44	50
Dominance D	0.2872	0.535	0.36	0.255	0.469	0.3096
Simpson 1-D	0.7128	0.465	0.64	0.745	0.531	0.6904
Shannon H	1.434	0.8711	1.139	1.51	1.106	1.276
Evenness e^∧^H/S	0.6989	0.5974	0.7808	0.7544	0.5038	0.7162
Sørensen index	0.60		0.2		0.182	

### Phylogenetic Analyses of Ophiostomatoid Isolates

A total of 366 DNA sequences (126 ITS; 84 TUB; 89 TEF; 67 CAL) were obtained to fully describe fungal communities in the non-infected and PWN-infected *P. pinaster* trees. All representative sequences for the four loci were BLAST against the NCBI GenBank database to retrieve the closest accessions (Supplementary Tables 2–5). From these sequences, only Ophiostomatales were considered for further phylogenetic analyses due to their close relation with *B. xylophilus* (Futai, 2013) (Table 3). Two datasets were considered based on preliminary BLAST results from ITS data: *Ophiostoma s. l*. and *Leptographium s. l*. as defined by de Beer et al. (2013). The amplification of ITS sequences for *Leptographium s. l*. species is often problematic (de Beer et al., 2013; Chang et al., 2017), and for some isolates were not obtained. In these cases, their taxonomic identification was supported by the other molecular markers. Additionally, the presence and absence of introns for TUB, TEF, and CAL sequences in both complexes, forced their separate analyses. Reference sequences for complexes within each dataset (*Ophiostoma s. l*. and *Leptographium s. l*.) were retrieved from GenBank (Supplementary Table 3).

**Table 3:** Fungal isolates representative of the main Ophiostomatales genus isolated in this study.

**Group taxon**	**Species**	**Isolate no**.	**PWD**	**Origin**	**GenBank Accession no**.			
					**ITS**	**TUB**	**TEF**	**CAL**
1	*Graphilbum* sp. 1	L2.2	PWD-symptomatic	Companhia das Lezírias	OM468578	–	–	–
	*Graphilbum* sp. 2	L4.2	PWD-symptomatic	Companhia das Lezírias	OK559549	–	OM616860	–
	*Graphilbum* sp. 3	L5.1	PWD-symptomatic	Companhia das Lezírias	OK559550	–	OM616861	–
	*Graphilbum* sp. 4	S38.7	PWD-symptomatic	Seia	–	–	–	–
2	*Leptographium terebrantis*	S38.3	PWD-symptomatic	Seia	OM468594	OM514986	–	–
	*Leptographium terebrantis*	S38.9	PWD-symptomatic	Seia	OM468595	ON333628	ON333659	ON333660
	*Leptographium terebrantis*	S40.4b	PWD-symptomatic	Seia	OK559544	OM514987	OM616854	–
3	*Leptographium sosnaicola*	L2.6	PWD-symptomatic	Companhia das Lezírias	OK559548	OM514991	OM616859	OM616866
4	*Leptographium* sp. 1	T2.13	PWD-symptomatic	Tróia	OK559547	OM514990	–	–
	*Leptographium* sp. 2	T7.1	PWD-symptomatic	Tróia	OM468585	ON333629	–	ON333661
	*Leptographium* sp. 3	S40.11	PWD-symptomatic	Seia	–	OM513989	OM616856	–
	*Leptographium* sp. 4	T3.1	PWD-symptomatic	Tróia	OK559545	OM514988	OM616857	OM616865
	*Leptographium* sp. 5	T3.6	PWD-symptomatic	Tróia	–	–	OM616858	–
5	*Ophiostoma ips*	L3.1	PWD-symptomatic	Companhia das Lezírias	OM468579	ON333612	ON333630	ON333647
	*Ophiostoma ips*	L4.1	PWD-symptomatic	Companhia das Lezírias	OM468580	ON333613	ON333631	–
	*Ophiostoma ips*	L5.2	PWD-symptomatic	Companhia das Lezírias	OM468581	ON333614	ON333632	–
	*Ophiostoma ips*	T1.4	PWD-symptomatic	Tróia	OM468583	ON333615	ON333633	ON333648
	*Ophiostoma ips*	T2.3	PWD-symptomatic	Tróia	OM468584	ON333616	ON333634	ON333649
	*Ophiostoma ips*	T7.4	PWD-symptomatic	Tróia	OM468586	ON333617	ON333635	ON333650
	*Ophiostoma ips*	S13.1	Non-symptomatic	Seia	OM468587	ON333618	ON333636	ON333651
	*Ophiostoma ips*	S19.2	Non-symptomatic	Seia	OM468588	ON333619	ON333637	ON333652
	*Ophiostoma ips*	S19.17	Non-symptomatic	Seia	OM468589	ON333620	ON333638	–
	*Ophiostoma ips*	S32.10	Non-symptomatic	Seia	OM468590	ON333621	ON333639	ON333653
	*Ophiostoma ips*	S35.1	PWD-symptomatic	Seia	OM468591	ON333622	ON333640	ON333654
	*Ophiostoma ips*	S35.3	PWD-symptomatic	Seia	OM468592	ON333623	ON333641	ON333655
	*Ophiostoma ips*	S36.1	PWD-symptomatic	Seia	OK559539	OM616846	OM616850	–
	*Ophiostoma ips*	S36.9	PWD-symptomatic	Seia	OM468593	ON333624	ON333642	ON333656
	*Ophiostoma ips*	S38.16	PWD-symptomatic	Seia	OK559541	OM616848	OM616853	OM616862
	*Ophiostoma ips*	S39.1	PWD-symptomatic	Seia	OK559540	OM616847	OM616851	–
	*Ophiostoma ips*	S39.6	PWD-symptomatic	Seia	OM468596	–	ON333643	–
	*Ophiostoma ips*	S40.10	PWD-symptomatic	Seia	OK559542	OM616849	OM616855	OM616863
	*Ophiostoma ips*	S40.1a	PWD-symptomatic	Seia	OM468597	ON333625	ON333644	ON333657
	*Ophiostoma ips*	S40.7	PWD-symptomatic	Seia	OM468598	ON333626	ON333645	ON333658
6	*Sporothrix* sp. 1	L5.3	PWD-symptomatic	Companhia das Lezírias	OM468583	ON333627	ON333646	–

#### Leptographium Sensu Lato

**Figure 2:**
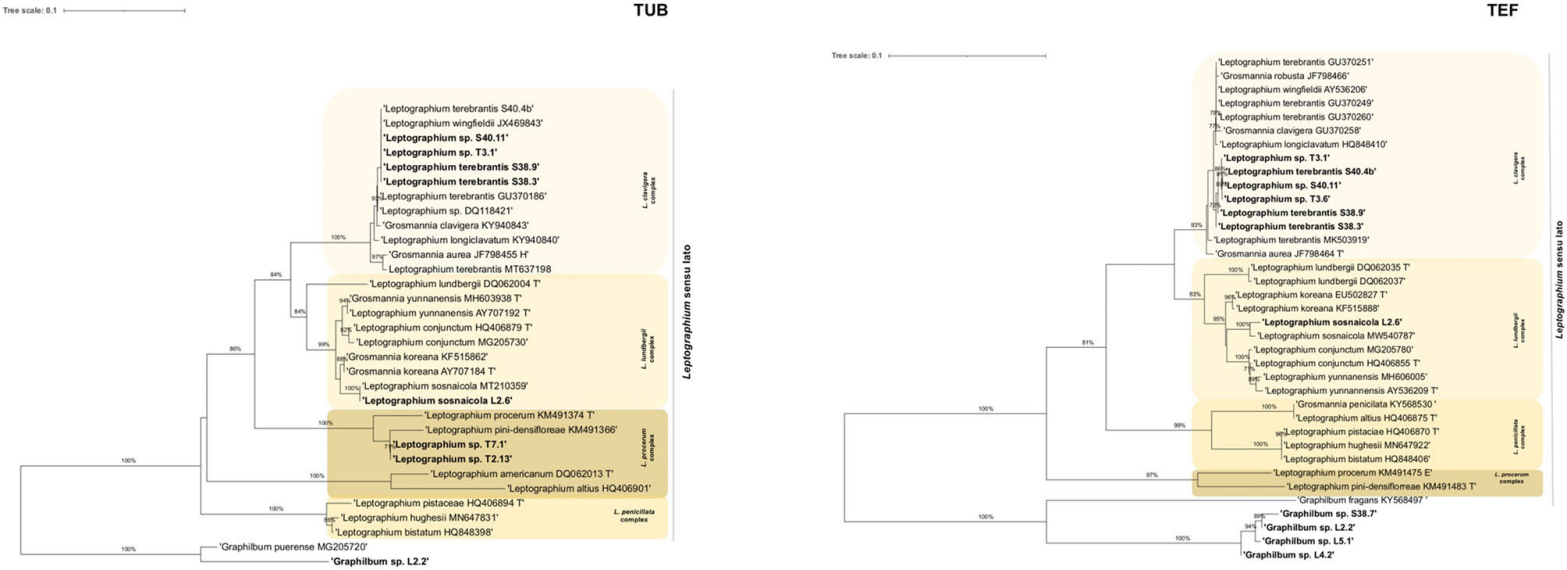
Maximum likelihood trees of *Leptographium sensu lato* generated by β-tubulin (TUB) and elongation factor 1-α (TEF) DNA sequence data. Sequences generated from this study are presented in bold (Table 2). Bootstrap values ≥70% are presented above the respective branch. Branch length is a measure of the number of substitutions per site (scale bar). T = ex-type isolates.

Within *Leptographium s. l*., four taxa were described: taxon 1, *Graphilbum* sp. 1-4; taxon 2, *Leptographium terebrantis*; taxon 3, *Leptographium* sp. 1-4; and taxon 4, *Leptographium sosnaicola* (Table 3). Multiple sequence alignment contained for each marker, respectively: 39 sequences with 665 bp, with gaps, for ITS; 31 sequences with 414 characters (with gaps) for TUB; 37 sequences with 528 bp (with gaps) for TEF; and 13 sequences with 597 bp (with gaps) for CAL (Figure 2, Supplementary Figures 3, 5). The best evolutionary substitution models for both trees were general time reversible (GTR) +G (rate variation) + T (topology variation). The *Leptographium s. l*. is well-represented by *L. clavigera, L. lundbergii, L. penicillate*, and the *L. procerum* complexes (Figure 2). The group taxon 1, *Graphilbum* sp., was considered the outgroup of all trees of *Leptographium s. l*. Within this group, *Graphilbum* sp. L 2.2 (TUB, TEF), *Graphilbum* sp. L4.2, L5.1, and S38.7 (TEF) clustered together with other *Graphilbum* species; however, their percentage of identity was lower than 98% (Supplementary Table 2), not allowing their species-level assignment. Taxon 2 and Taxon 3 belonged to *L. clavigera* complex (in both markers) and were grouped in-between *L. wingfieldii* and *L. terebrantis* with high support. In fact, isolates S38.3/S38.9 (isolated from the same tree) and S40.4b shared 100% similarity with *L. terebrantis*, while the other isolates (T3.1, T3.6, S40.11) were 100% identical in TUB but 99% in TEF (Supplementary Table 2). In CAL sequences, these isolates were identified as *Grosmannia clavigera* (100%) by the lack of *Leptographium* sequences in the database (Supplementary Table 2). Taxon 4 *L. sosnaicola* L2.6 formed a monophyletic group with *L. sosnaicola* MT210359 (TUB) and *L. sosnaicola* MW540787 (TEF), with good support, and clustering in between *L. koreana* and *L. conjunctum* within the *L. lundbergii* complex.

#### Ophiostoma Sensu Lato

**Figure 3:**
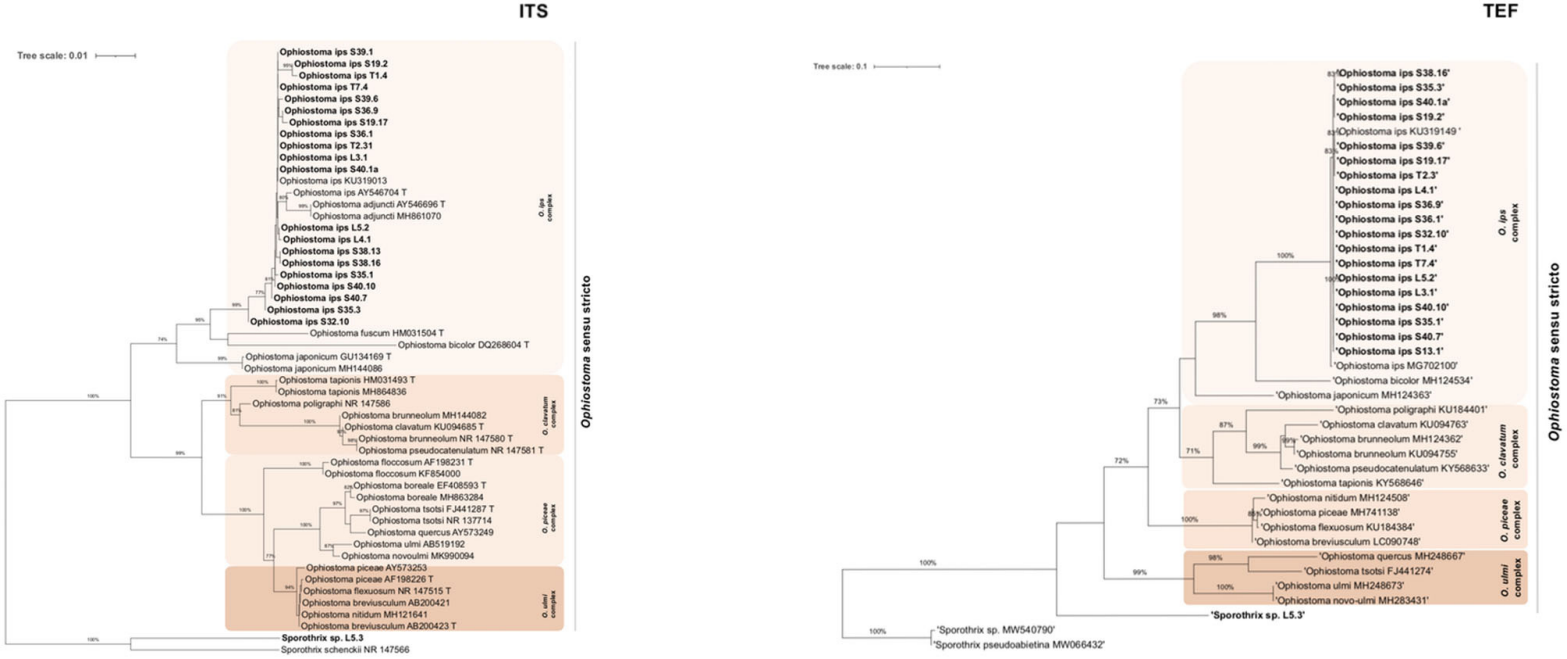
Maximum likelihood trees of *Ophiostoma sensu stricto* generated by internal-transcribed spacer (ITS2) and elongation factor 1-α (TEF) DNA sequence data. Sequences generated from this study are presented in Bold (Table 2). Bootstrap values ≥70% are presented above the respective branch. Branch length is a measure of the number of substitutions per site (scale bar). T = ex-type isolates.

Within this lineage, only two taxa were described: taxon 5, *Ophiostoma ips* and taxon 6, *Sporothrix* sp.1 (Table 3). The alignment matrixes contained, respectively, 48 sequences (Table 2, Supplementary Table 3) with 648 characters, including gaps, for ITS marker; 43 sequences with 220 bp for TUB marker; 40 sequences with 379 bp for TEF; and 29 sequences with 339 bp for CAL (Figure 3, Supplementary Figures 4, 5). The best evolutionary substitution models for ITS and TEF phylograms were, respectively, Hasegawa-Kishino-Yano (HKY) +G+T and GTR+G+T. The well-defined phylogenetic complexes with *Ophiostoma s. str*. (*O. ips* complex, *O. piceae* complex, *O. ulmi* complex, and *O. clavatum* complex) were maintained, when possible, for all phylogenetic trees of *Ophiostoma s. l*. Fungal isolates from all locations and tree status, previously identified as *O. ips*, clustered together with good support with the type strain *O. ips* AY546704 and *O. ips* KU319013 (described in *P. sylvestris* in Poland) within the *O. ips* complex (Figure 3). Similar topologies were obtained in ML phylogenetic trees with the other molecular markers (BT, TEF and CAL; Figure 3, Supplementary Figures 4, 5), which sustained the identification of *O. ips* fungal isolates. Belonging to *Ophiostoma s. l*., the isolate L5.3 was identified as *Sporothrix* sp. 1 (99% similarity with *Sporothrix* sp. MW540762.1 with ITS; 100% *S. pseudoabietina* MH583598 with TUB; and 99.39% *Sporothrix* sp. KY568667 with TEF; Supplementary Table 2). In Figure 3, this isolate clustered with good support with *S. schenckii* NR_147566, being considered as the outgroup of this phylogenetic tree. Its position is also corroborated in Supplementary Figures 4, 5.

### Adaptative Responses of Ophiostomatoid Isolates to Temperature, Antagonism, and PWN Interaction

**Figure 4:**
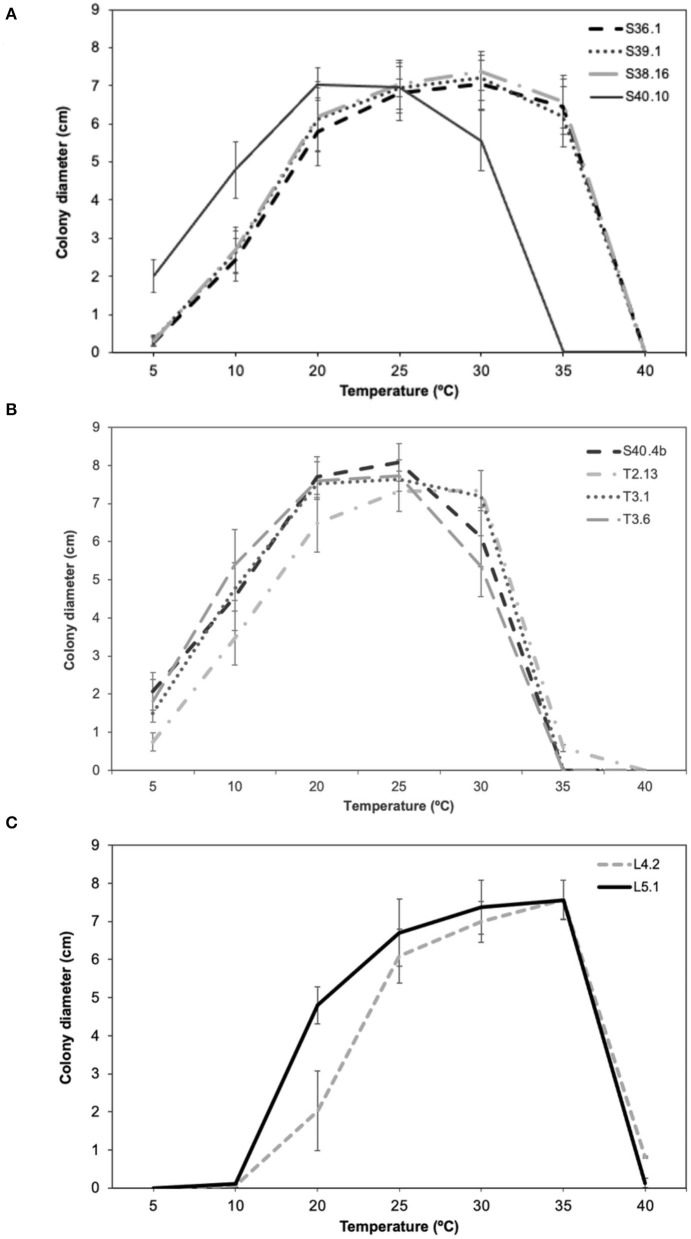
Comparison of mean growth per day (± standard error) of **(A)** *Ophiostoma ips* (S36.1, S39.1, S38.16, S40.10), **(B)** *Leptographium* sp. (S40.4b, T2.13, T3.1, T3.6), and **(C)** *Graphilbum* sp. (L4.2, L5.1).

To characterize the adaptative response (in terms of growth and antagonistic performance and PWN food source) of the main Ophiostomatales taxon (genus *Ophiostoma, Leptographium*, and *Graphilbum*) isolated from PWN-infected *P. pinaster*, ten ophiostomatoid fungal isolates (S36.1; S39.1; S38.16; S40.4b; S40.10; T2.13; T3.1; T3.6; L4.2; and L5.1) were selected as representatives. Only one isolate of *Sporothrix* was obtained and therefore was not included in these trials. Since the collection sites presented different geographic and climate conditions (in terms of average temperature and precipitation; Table 1), the selected fungal isolates were tested in a wide amplitude of temperatures between 5 and 40°C during 15 days of incubation (Figure 4). Overall, almost all fungal isolates could grow between 20 and 35°C; however with different optimal temperatures. All taxa failed to grow at 40°C. For *Ophiostoma* group (Figure 4A), the optimal growth temperature varied between 20°C (for S40.10) and 30°C (for S36.1, S39.1, S38.16). At lower temperatures (5–10°C), only S40.10 exhibited discrete growth but failed to grow at 35°C. For *Leptographium* group (Figure 4B), the optimal growth temperature ranged between 25°C (S40.4b, T3.1, and T3.6) and 30°C (T2.13). For *Graphilbum* sp. L4.2 and L5.1, the optimal growth temperature was 35°C (Figure 4C), yet these isolates were unable to grow at 5 or 10°C.

**Figure 5:**
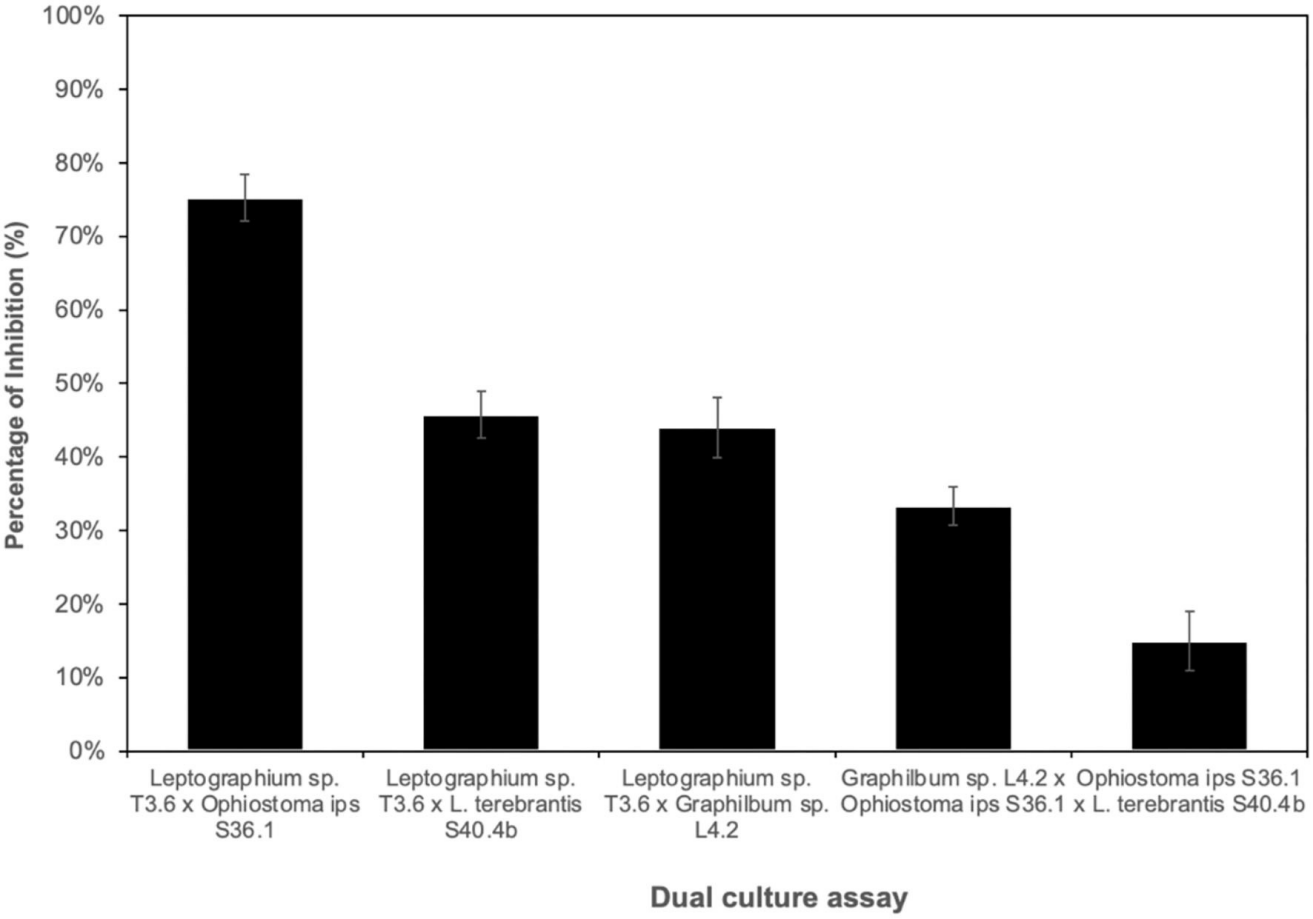
Percentage of inhibition of the potential antagonist x tested organism (grown at 25°C for 5 days).

For antagonistic performance of ophiostomatoid isolates, different combinations between representatives of the main taxa were tested considering a potential antagonist (R1) and a test organism (R2) (Supplementary Figure 6). Overall, the fungal isolate *Leptographium sp*. T3.6 inhibited the growth of the other 3 isolates [*O. ips* S36.1; *Graphilbum* sp. L4.2; *L. terebrantis* S40.4b (Figure 5)]. The percentage of inhibition of *Leptographium* sp. T3.6 and *Ophiostoma ips* S36.1 was 75%, while for *Graphilbum* sp. L4.2 and *L. terebrantis* S40.4b, they were 44 and 46%, respectively. No differences were seen between *Leptographium* sp. T3.6 and *L. terebrantis* S40.4b or *Graphilbum* sp. L4.2. The dual culture of *Graphilbum* sp. L4.2 x *O. ips* S36.1 and *O. ips* S36.1 x *L. terebrantis* S40.4b recorded the lowest inhibition, respectively, 33 and 15%.

**Figure 6:**
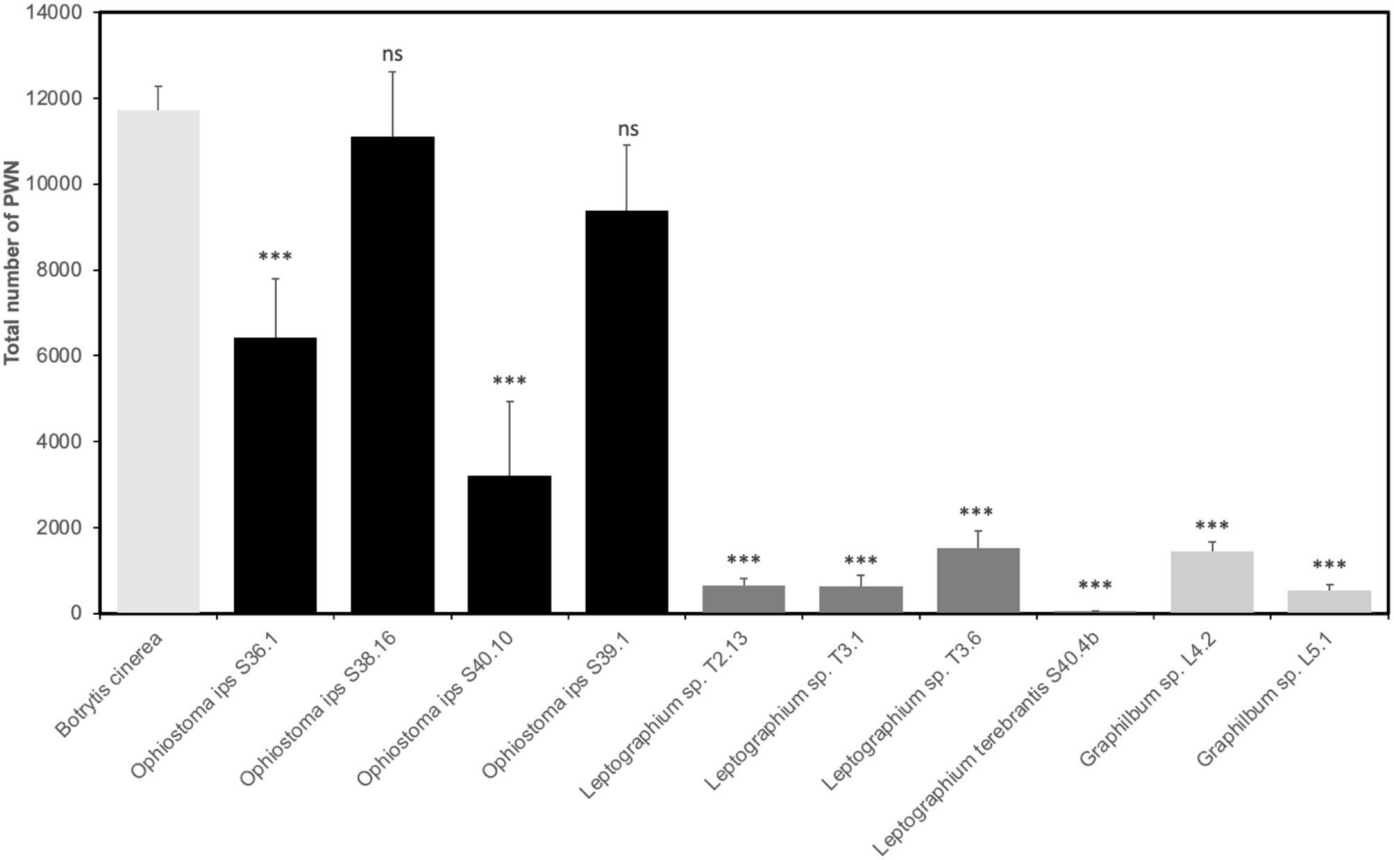
Reproduction of *Bursaphelenchus xylophilus* population in each ophiostomatoid fungi and *Botrytis cinerea* (control) (mean total no. nematodes ± standard error). Asterisks (***) indicate statistical differences at 99% of confidence in relation to *B. cinerea*.

Not all Ophiostomatales could support PWN growth. Except for *O. ips* S38.16 and S39.1, all fungal isolates tested were unsuitable for PWN as the food source (*p* < 0.001), when comparing with *B. cinerea* (total number of PWN after 7 days was 11,733 ± 546) (Figure 6). Within the group of *O. ips*, isolate S40.10 recorded a population increase of 27.4% when compared with the control, while S36.1 and S39.1 were, respectively, 54.8 and 80%. *Ophiostoma ips* S38.16 showed a close result with *B. cinerea*, with 94.6% of PWN growth (*p* > 0.001). For the *Leptographium* group, PWN growth was relatively low (<13%). In fact, PWN could not grow completely in S40.4b. The total number of PWN recovered from *Leptographium* sp. T2.13 and T3.1 were, respectively, 649 ± 160 and 636 ± 251 nematodes (~5.5% in comparison with *B. cinerea*). The two isolates of *Graphilbum* tested, L4.2 and L5.1, were also not suitable for PWN, with 12 and 4.5%, respectively, of PWN growth in comparison with the control fungus.

## Discussion

Fungal communities seem to dominate different ecological niches in forest tree habitats due to their easy adaptation to wood colonization (de Boer et al., 2005). In this sense, these communities are more likely to undergo more severe shifts in abundance and composition due to biotic and abiotic stresses (Baldrian, 2017). The study of the fungal communities in the PWD complex, and their intermediates, are still underrated (Zhao et al., 2014; Nascimento et al., 2015) in spite of their strong influence on PWN populations (Vicente et al., 2021). In this study, we present a detailed characterization of fungal communities from non-infected and PWN-infected *P. pinaster* trees from three contrasting PWD foci (long-term PWN presence, Companhia das Lezírias and Tróia, and transitional/recent PWN detection, Seia). This is the first study focused exclusively on the fungal diversity in PWD affected areas, in Europe. In addition, we also characterized the adaptative response of a specific group of Ophiostomatales fungi (genus *Ophiostoma, Leptographium, Graphilbum*) due to their well-recognized history with PWN (Vicente et al., 2021), as means to uncover their resilience in the disease complex.

### Mycoflora Communities in the PWD

The surveys were conducted during early winter 2019 and spring 2020 before the maturation feeding of the insect-vector, which could justify the high PWN numbers extracted from the sampled wood. Despite the discrepancy in the total number of fungal isolates between sampling locations, overall, we observed clear differences in the fungal communities between collection sites, being long-term PWD loci (Companhia das Lezírias and Tróia) closest between themselves than each with the most recent PWD area (Seia). Several factors may influence this result, e.g., their PWD history or biogeographical conditions of the locations, which are completely distinct in terms of temperature/precipitation/altitude, as well as seasonality. In Seia, the fungal communities of non-infected and PWN-infected *P. pinaster* showed substantial differences in terms of composition and species richness, the non-infected pine trees being more biodiverse than PWN-infected trees. This observation suggests that the presence of the nematode could explain the community shifts which is corroborated by recent works with other *Pinus* species (Liu et al., 2021; Zhang et al., 2021). Still, this result is not evident for fungal communities in Companhia das Lezírias or Tróia, according to the diversity indexes. The fact that these locations have a long-term history of PWN presence could be an indication of communities' stability in the presence of the nematode invader. Kuroda and Ito (1992) also reported similar species in healthy and wilted *P. thunbergii*, which varied vaguely among seasons. Zhao et al. (2013) also suggested a link between disease history and microbial communities' diversity; however only infected trees were used in this study. In our work, most of the taxa observed were already reported for other *Pinus* species, as well as PWN and insect-vector *Monochamus* sp. (Hyun et al., 2007; Zhao et al., 2013, 2018). However, with the exception of two *P. pinaster* trees from Seia, only PWN-infected *P. pinaster* trees were colonized by Ophiostomatales, the causal agents of blue-stain (Six, 2012) commonly present in pine trees infected by the PWN (Futai, 2013). The most dominant ophiostomatoid species was *O. ips*. This species was firstly reported in association with the infection of bark beetles in south-eastern North America (Rumbold, 1931), and nowadays is distributed worldwide. In PWD complex, *O. ips* was described as associated with PWN-infected *P. thunbergii*, PWN, and *M. alternatus* in Korea (Hyun et al., 2007) in *P*. *massoniana* and *P. thunbergii* in China (Zhao et al., 2013, 2018; Wang et al., 2018), and *M. galloprovincialis* and *P. pinaster* in Portugal (Inácio et al., 2015; Trindade, 2019). As suggested by Wang et al. (2018), the dominance of *O. ips* can have either an indigenous origin or be an effective adaptation to local pine forests. In fact, the presence of *O. ips* in non-infected trees in Seia could be explained by natural infection with various species of bark beetles as is common in European pine forests (Chang et al., 2017; Jankowiak et al., 2021). The other ophiostomatoid isolates were *Leptographium* sp., *Graphilbum* sp., and *Sporothrix* sp., which were also described in other studies (Zhao et al., 2013, 2018; Wang et al., 2018). For the first time, we report *Leptographium* species associated with PWN-infected *P. pinaster* trees in Portugal, namely *L. sosnaicola* and *L. terebrantis*. *Leptographium sosnaicola* was described in logs of *P. sylvestris* in Poland and probably vectored by *Ips sexdentatus* (Jankowiak et al., 2021). The authors showed that *L. sosnaicola* formed a monophyletic line within other members of *Leptographium*, and its morphology did not resemble any known *Leptographium* species. Our results also show *L. sosnaicola* L2.6 within *L. lundburgii* complex and totally separated from the other sister species, corroborating the previous observations (Jankowiak et al., 2021). The other *Leptographium* species identified *L. terebrantis*, firstly described as associated with the black turpentine beetle *Dendroctonus terebrans* (Barras and Perry, 1971), was reported as pathogen of woody roots and recently related to crown symptoms and tree mortality in *P. taeda* (Mensah et al., 2021). Hausner et al. (2005) showed that ex-type culture of *L. terebrantis* is very similar morphologically *to L. wingfieldii*, which explains their clustering and non-resolved placement in the phylogenetic trees of this study. Although only a few isolates were recovered from PWN-infected *P. pinaster, Graphilbum* and *Sporothrix* genus are common in pine trees infected with the nematode. Zhao et al. (2013) showed that *Sporothrix* sp. 1 could sustain PWN growth with female-biased sex ratio offspring and suggested that the prevalence of this species was correlated with higher tree mortality that possibly influenced the nematode and insect-vector populations in the area studied. This ophiostomatoid fungi was also isolated in South Korea (Hyun et al., 2007) and other regions of China (Wang et al., 2018). As for other *Leptographium* isolates in this study, neither morphological characterization nor molecular identification was resolved enough to assign it into species level, which will be explored in future studies for the description of new species.

### Ophiostomatales Adaptation and Interaction With PWN

The presence of the PWN induces a disturbance in microbial (bacteria and fungi) community stability, which has been already analyzed using metagenomics approaches (Alves et al., 2016; Liu et al., 2021). Ecologically, this is often related with community insensitivity (resistance) or the rate at which community returns to a pre-disturbance condition, intrinsically related to their functional role in the ecosystem (Shade et al., 2012). In this study, the isolates of *Ophiostoma, Leptographium*, and *Graphilbum* groups showed differences in temperature adaptation, which may reflect the environmental conditions of their collection sites. The *O. ips* group grew in lower temperatures such as 5–10°C, which is characteristic of the Northern areas. *Leptographium* sp. isolates and *Graphilbum* sp. isolates showed a preference for higher temperatures, with an optimal growth at 25 and 35°C, respectively. Zhao et al. (2018) surveyed distinct regions of China forests and detected *O. ips* mainly in the Southern sites, with higher annual temperatures (16°C) and rainfall (1,020–1,408 mm). Cobian et al. (2019) demonstrated that plant-microbe specificity varies with different abiotic parameters such as temperature or elevation. They also showed a strong interaction between host and fungal endophyte, and that the environment plays a secondary role in fungal communities, mostly by modulating host specialization (Cobian et al., 2019). Overall, it seems that associations between ophiostomatoid fungi and the pine species in the context of PWD may be variable, suggesting a co-evolution history, however further studies are needed to address this issue.

Competitive interactions among different fungal symbionts of bark-beetles (such as *O. minus, C. ranaculosis*, and *Entomocorticium* sp.) have been reported in artificial and natural conditions (Klepzig and Hofstetter, 2011). Klepzig (1998) showed that on an artificial medium the biological control fungus *O. piliferum* outcompete all three fungi (*O. minus, C. ranaculosis*, and *Entomocorticium* sp.); however, in inoculations of natural substrate, *O. piliferum* compete successfully with the two mutualistic fungi *C. ranaculosis* and *Entomocorticium* sp., but not with *O. minus*. Here, we evaluated pairwise competition between the three main taxa and found that *Leptographium* sp. could outcompete neither with *Ophiostoma* nor with *Graphilbum*, suggesting potential antagonism between Ophiostomatales. Considering our sampled trees, this result could also explain why only few *O. ips* were collected in the trees mainly dominated with *Leptographium*. So far, we could not find any other study suggesting this kind of ecological interactions between ophiostomatoid fungi in the context of PWD.

Fukushige (1991a,b) showed that *B. xylophilus* propagation on fungus could differ in agar media and pine wood. The colonization of *O. minus* in pine-branch segments of *P. densiflora* resulted in a quick increase of PWN population (Maehara and Futai, 2000). Zhao et al. (2018) showed that *L. pini-densiflorae* could promote the highest population density, followed by *Sporothrix* sp. 1, while *O. ips* or *O. minus* resulted in significantly lower PWN populations. Pimentel et al. (2021) evaluated nematode propagation in 10 different species of Ascomycota fungi, including different Ophiostomales species (*O. ips, O. piliferum, O. minus*) and *Leptographium* (*L. procerum, L. terebrantis*). After 20 days of incubation, the nematode propagation in these fungal isolates was unexcepted, since all were unsuitable for PWN growth (Pimentel et al., 2021). In this sense, not all ophiostomatoid fungi have been considered suitable for PWN growth in declining trees (Zhao et al., 2014). In this study, the highest PWN multiplication in comparison with their *B. cinerea* culture fungi was obtained in the *O. ips* group, while *Leptographium* and *Graphilbum* group resulted in significantly lower PWN numbers in the conditions tested. Intraspecific variability within the *O. ips* group was also seen. With the exception of *O. ips* S40.10, all the other isolates had a positive impact on the PWN population, which seems to be in line with other studies (Maehara and Futai, 1997; Maehara, 2008). Indeed, this pattern seemed to be sustained by the initial quantification of PWN-infected trees. In Seia site, where *O. ips* was the most prevalent ophiostomatoid fungi (almost 70% of community composition, 3 out of the 5 pine trees presented a population of PWN between 1,001 and 5,000 nematodes per 100 g of wood (class IV). The Tróia site evidenced low to medium levels of PWN infection. In the specific case of one tree (T3), classified as class II (51-200 nematodes per 100 g of wood), the low concentration of PWN could be explained by the mycoflora present, given that all isolated fungi belonged to the *Leptographium* genus, which could not support PWN multiplication in the feeding trials. As for the two *Graphilbum* sp. isolates, the defoliation class of the trees sampled were class III and IV, not supporting the previous observations. Still, this seemingly relation between the host defoliation class and the impact of their isolated fungi in PWN multiplication is worth pursuing, especially confirming their effect in the native host *P. pinaster*. The present research constitutes a milestone in the study of the functional role of fungal communities inhabiting wilted trees, their ecological interactions, and their suitability for PWN growth, as well as their impact on PWD expression. This knowledge can lead to the identification of key points in PWN life cycle that can be targeted to disrupt the disease cycle using more natural alternatives.

## Data Availability Statement

The datasets presented in this study can be found in online repositories. The names of the repository/repositories and accession number(s) can be found in the article/Supplementary Material.

## Author Contributions

CV and MI: conceptualization. CV, MS, AR, and ME: research and data analysis. CV and JF: writing—original draft preparation. ME, AR, MM, FN, and MI: writing—review and editing. AR, MM, and MI: resources. All authors contributed to the article and approved the submitted version.

## Funding

This research was funded by the Fundação para a Ciência e Tecnologia (FCT) and Fundo Europeu de Desenvolvimento Regional (FEDER) within the Programa Operacional Regional de Lisboa and Programa Operacional Regional do Alentejo through the national project LISBOA-01-0145-FEDER028724 - PTDC/ASP-PLA/28724/2017 (ALT20-03-0145-FEDER-028724) PineENEMY, Exploring the NEmatode-MYcobiota interactions in Pine Wilt Disease, through the R&D Unit, UIB/04551/2020 (GREEN-IT - Bioresources for Sustainability), and the CEECIND/00040/2018 (to CV).

## Conflict of Interest

The authors declare that the research was conducted in the absence of any commercial or financial relationships that could be construed as a potential conflict of interest.

## Publisher's Note

All claims expressed in this article are solely those of the authors and do not necessarily represent those of their affiliated organizations, or those of the publisher, the editors and the reviewers. Any product that may be evaluated in this article, or claim that may be made by its manufacturer, is not guaranteed or endorsed by the publisher.
