# Supplementary material for: Fungal Communities of the Pine Wilt Disease Complex: Studying the Interaction of Ophiostomatales With Bursaphelenchus xylophilus
Source: Front Plant Sci. 2022 Jun 14;13:908308. doi: 10.3389/fpls.2022.908308 (PMC9257700; doi:10.3389/fpls.2022.908308)
Supplement: Supplementary file 1 [file Table_1.DOCX]

Supplementary Material

**Supplementary Table 1** – List of primers used in this study.

| **Gene Marker** | **Primer Name** | **Primer sequence 5' - 3'** | **Reference** |
| --- | --- | --- | --- |
| Internal Transcribed Spacer (ITS) | ITS1f | CTTGGTCATTTAGAGGAAGTAA | Gardes and Bruns, 1993 |
|  | ITS4 | TCCTCCGCTTATTGATATGC | White et al. 1990 |
| Beta-tubulin (TUB | BT2a | GGTAACCAAATCGGTGCTGCTTTC | Glass and Donaldson, 1995 |
|  | BT2b | ACCCTCAGTGTAGTGACCCTTGGC |  |
| Calmodulin (CAL) | CL1 | GARTWCAAGGAGGCCTTCTC | O'Donnell, 2000 |
|  | CL2A | TTTTTGCATCATGAGTTGGAC |  |
| Calmodulin (CAL) | CL2F | GACAAGGAYGGYGATGGT | Duong et al., 2012 |
|  | CL2R | TTCTGCATCATGAGYTGSAC |  |
| Elongation factor 1-alpha (TEF) | EF-728F | CATCGAGAAGTTCGAGAAGG | Carbone and Kohn, 1999 |
|  | EF-2 | GGARGTACCAGTSATCATGTT | Jacobs et al., 2004 |

**Supplementary Table 2** – Best BLAST hit of each fungal isolate based upon ITS partial sequence retrieved from the NCBI GenBank database.

| **Isolate ID** | **Tree ID** | **Origin** | ***Pinus pinaster* symptoms** | **BLAST Nucleotide** | | | | |
| --- | --- | --- | --- | --- | --- | --- | --- | --- |
|  |  |  |  | **Description** | **Accession** | **Identity (%)** | **E-value** |  |
| L2.1 | L2 | Companhia das Lezirias | PWD-symptomatic | *Graphilbum acuminatum* | MN548899.1 | 97.06 | 0.0 |  |
| L2.2 | L2 | Companhia das Lezirias | PWD-symptomatic | *Graphilbum acuminatum* | MN548899.1 | 97.06 | 0.0 |  |
| L2.3 | L2 | Companhia das Lezirias | PWD-symptomatic | *Graphilbum acuminatum* | MN548899.1 | 97.22 | 0.0 |  |
| L2.5 | L2 | Companhia das Lezirias | PWD-symptomatic | *Graphilbum acuminatum* | MN548899.1 | 96.85 | 0.0 |  |
| L2.6 | L2 | Companhia das Lezirias | PWD-symptomatic | *Leptographium sosnaicola T* | NR_172302.1 | 99.84 | 0.0 |  |
| L3.1 | L3 | Companhia das Lezirias | PWD-symptomatic | *Ophiostoma ips* | MH324814.1 | 99.70 | 0.0 |  |
| L3.2 | L3 | Companhia das Lezirias | PWD-symptomatic | *Ophiostoma ips* | MH324814.1 | 99.69 | 0.0 |  |
| L3.3 | L3 | Companhia das Lezirias | PWD-symptomatic | *Ophiostoma ips* | MH324814.1 | 99.85 | 0.0 |  |
| L4.1 | L4 | Companhia das Lezirias | PWD-symptomatic | *Ophiostoma ips* | MH324814.1 | 99.85 | 0.0 |  |
| L4.2 | L4 | Companhia das Lezirias | PWD-symptomatic | *Graphilbum acuminatum* | MN548899.1 | 97.06 | 0.0 |  |
| L4.12 | L4 | Companhia das Lezirias | PWD-symptomatic | *Arcopilus sp.* | MH619611.1 | 100 | 0.0 |  |
| L5.1 | L5 | Companhia das Lezirias | PWD-symptomatic | *Graphilbum acuminatum* | MN548899.1 | 97.27 | 0.0 |  |
| L5.2 | L5 | Companhia das Lezirias | PWD-symptomatic | *Ophiostoma ips* | MH324814.1 | 99.69 | 0.0 |  |
| L5.3 | L5 | Companhia das Lezirias | PWD-symptomatic | *Sporothrix sp.* | MW540762.1 | 99 | 0.0 |  |
| T1.2 | T1 | Tróia | PWD-symptomatic | *Ophiostoma ips* | OK559539.1 | 100 | 0.0 |  |
| T1.3 | T1 | Tróia | PWD-symptomatic | *Ophiostoma ips* | MW046089.1 | 99.84 | 0.0 |  |
| T1.4 | T1 | Tróia | PWD-symptomatic | *Ophiostoma ips* | OK559539.1 | 100 | 0.0 |  |
| T1.5 | T1 | Tróia | PWD-symptomatic | *Ophiostoma ips* | OK559539.1 | 100 | 0.0 |  |
| T1.9 | T1 | Tróia | PWD-symptomatic | *Ophiostoma ips* | OK559539.1 | 100 | 0.0 |  |
| T1.10 | T1 | Tróia | PWD-symptomatic | *Ophiostoma ips* | OK559539.1 | 100 | 0.0 |  |
| T1.14 | T1 | Tróia | PWD-symptomatic | *Backusella lamprospora* | MH854973.1 | 99.87 | 0.0 |  |
| T1.15 | T1 | Tróia | PWD-symptomatic | *Fusarium babinda* | MT478951.1 | 99.80 | 0.0 |  |
| T1.16 | T1 | Tróia | PWD-symptomatic | *Backusella lamprospora* | MH854973.1 | 99.87 | 0.0 |  |
| T1.17 | T1 | Tróia | PWD-symptomatic | *Ophiostoma ips* | MH324814.1 | 100 | 0.0 |  |
| T2.3 | T1 | Tróia | PWD-symptomatic | *Ophiostoma ips* | MH323814.1 | 100 | 0.0 |  |
| T2.4 | T2 | Tróia | PWD-symptomatic | *Ophiostoma ips* | OK559539.1 | 100 | 0.0 |  |
| T2.5 | T2 | Tróia | PWD-symptomatic | *Ophiostoma ips* | MH323814.1 | 100 | 0.0 |  |
| T2.13 | T2 | Tróia | PWD-symptomatic | *Leptographium procerum* | JX444636.1 | 98.99 | 1.2E-144 |  |
| T2.18 | T2 | Tróia | PWD-symptomatic | *Ophiostoma ips* | OK559539.1 | 100 | 0.0 |  |
| T3.1 | T3 | Tróia | PWD-symptomatic | *Leptographium wingfieldii* | JX444639.1 | 100 | 0.0 |  |
| T3.2 | T3 | Tróia | PWD-symptomatic | *Leptographium wingfieldii* | JX444639.1 | 100 | 0.0 |  |
| T3.3 | T3 | Tróia | PWD-symptomatic | *Leptographium wingfieldii* | OK559539.1 | 100 | 0.0 |  |
| T3.5 | T3 | Tróia | PWD-symptomatic | *Leptographium wingfieldii* | KP691916.1 | 100 | 0.0 |  |
| T3.6 | T3 | Tróia | PWD-symptomatic | *Leptographium* wingfieldii | JX444639.1 | 100 | 0.0 |  |
| T3.7 | T3 | Tróia | PWD-symptomatic | *Ophiostoma ips* | OK559539.1 | 100 | 0.0 |  |
| T3.8 | T3 | Tróia | PWD-symptomatic | *Leptographium* wingfieldii | JX444639.1 | 100 | 0.0 |  |
| T3.9 | T3 | Tróia | PWD-symptomatic | *Leptographium* wingfieldii | JX444639.1 | 100 | 0.0 |  |
| T7.1 | T7 | Tróia | PWD-symptomatic | *Leptographium procerum* | JX444636.1 | 100 | 6.38E-12 |  |
| T7.2 | T7 | Tróia | PWD-symptomatic | *Ophiostoma ips* | OK559539.1 | 100 | 0.0 |  |
| T7.3 | T7 | Tróia | PWD-symptomatic | *Ophiostoma ips* | OK559539.1 | 100 | 0.0 |  |
| T7.4 | T7 | Tróia | PWD-symptomatic | *Ophiostoma ips* | MH323814.1 | 100 | 0.0 |  |
| T7.5 | T7 | Tróia | PWD-symptomatic | *Ophiostoma ips* | OK559539.1 | 100 | 0.0 |  |
| T7.6 | T7 | Tróia | PWD-symptomatic | *Ophiostoma ips* | OK559539.1 | 100 | 0.0 |  |
| T7.7 | T7 | Tróia | PWD-symptomatic | *Ophiostoma ips* | OK559539.1 | 100 | 0.0 |  |
| T7.8 | T7 | Tróia | PWD-symptomatic | *Ophiostoma ips* | MH323814.1 | 100 | 0.0 |  |
| T8.1 | T8 | Tróia | Non-symptomatic | *Fusarium babinda* | MT478951.1 | 100 | 0.0 |  |
| T8.2 | T8 | Tróia | Non-symptomatic | *Fusarium babinda* | MT478951.1 | 100 | 0.0 |  |
| T8.3 | T8 | Tróia | Non-symptomatic | *Fusarium merismoides* | MK577888.1 | 100 | 0.0 |  |
| T8.7 | T8 | Tróia | Non-symptomatic | *Fusarium babinda* | MT478951.1 | 100 | 0.0 |  |
| T8.8 | T8 | Tróia | Non-symptomatic | *Nemania sp.* | MT153669.1 | 99.81 | 0.0 |  |
| T8.9 | T8 | Tróia | Non-symptomatic | *Umbelopsis isabellina* | HQ630365.1 | 99.83 | 0.0 |  |
| T8.12 | T8 | Tróia | Non-symptomatic | *Fusarium babinda* | MT478951.1 | 100 | 0.0 |  |
| T9.2 | T8 | Tróia | Non-symptomatic | *Fusarium babinda* | MW764919.1 | 100 | 0.0 |  |
| S1.3 | S1 | Seia | Non-symptomatic | *Alternaria brassicicola i* | MT635275 | 100.00 | 0.00 |  |
| S1.4 | S1 | Seia | Non-symptomatic | *Uncultured Alternaria* | JQ346879 | 100.00 | 0.00 |  |
| S1.5 | S1 | Seia | Non-symptomatic | *Trichoderma gamsii* | KX343108 | 100.00 | 0.00 |  |
| S1.6 | S1 | Seia | Non-symptomatic | *Alternaria sp.* | OM262356 | 100.00 | 0.00 |  |
| S1.7 | S1 | Seia | Non-symptomatic | *Alternaria brassicicola* | MT635275 | 100.00 | 0.00 |  |
| S2.1 | S2 | Seia | Non-symptomatic | *Penicillium citreonigrum* | AY373908 | 100.00 | 0.00 |  |
| S2.6 | S2 | Seia | Non-symptomatic | *Alternaria brassicicola* | MT635275 | 100.00 | 0.00 |  |
| S2.7 | S2 | Seia | Non-symptomatic | *Penicillium sp.* | MK027053 | 100.00 | 0.00 |  |
| S2.9 | S2 | Seia | Non-symptomatic | *Penicillium miczynskii* | NR_077156 | 100.00 | 0.00 |  |
| S9.1 | S9 | Seia | Non-symptomatic | *Trichoderma erinaceum* | MK862245 | 98.26 | 0.00 |  |
| S9.3 | S9 | Seia | Non-symptomatic | *Penicillium sp.* | KX869945 | 100.00 | 0.00 |  |
| S9.4 | S9 | Seia | Non-symptomatic | *Penicillium citreonigrum* | AY373908 | 100.00 | 0.00 |  |
| S13.1 | S13 | Seia | Non-symptomatic | *Ophiostoma ips* | OK559539 | 96.23 | 0.00 |  |
| S19.1 | S19 | Seia | Non-symptomatic | *Fusarium odoratissimum* | MW016597 | 100.00 | 0.00 |  |
| S19.2 | S19 | Seia | Non-symptomatic | *Ophiostoma ips* | AY172021 | 100.00 | 0.00 |  |
| S19.4 | S19 | Seia | Non-symptomatic | *Alternaria alternata* | MT174141 | 100.00 | 0.00 |  |
| S19.13 | S19 | Seia | Non-symptomatic | *Alternaria alternata* | MT453271 | 100.00 | 0.00 |  |
| S19.15 | S19 | Seia | Non-symptomatic | *Ophiostoma ips* | OK559542 | 100.00 | 0.00 |  |
| S19.16 | S19 | Seia | Non-symptomatic | *Ophiostoma ips* | AY172021 | 100.00 | 0.00 |  |
| S19.17 | S19 | Seia | Non-symptomatic | *Ophiostoma ips* | GU170426 | 100.00 | 0.00 |  |
| S19.19 | S19 | Seia | Non-symptomatic | *Ophiostoma ips* | OK104009 | 99.79 | 0.00 |  |
| S32.3 | S32 | Seia | Non-symptomatic | *Trichoderma atroviride* | MN795754 | 99.68 | 3.30E-157 |  |
| S32.4 | S32 | Seia | Non-symptomatic | *Trichoderma sp.* | MK871201 | 100.00 | 0.00 |  |
| S32.6 | S32 | Seia | Non-symptomatic | *Alternaria alternata* | MH170880 | 100.00 | 0.00 |  |
| S32.7 | S32 | Seia | Non-symptomatic | *Alternaria alternata* | MH170880 | 100.00 | 0.00 |  |
| S32.8 | S32 | Seia | Non-symptomatic | *Alternaria alternata* | MH521178 | 99.82 | 0.00 |  |
| S32.9 | S32 | Seia | Non-symptomatic | *Alternaria alternata* | MH170880 | 100.00 | 0.00 |  |
| S32.10 | S32 | Seia | Non-symptomatic | *Uncultured Ophiostoma sp.* | KU663983 | 100.00 | 0.00 |  |
| S32.12 | S32 | Seia | Non-symptomatic | *Alternaria sp.* | MK640592 | 99.64 | 0.00 |  |
| S35.1 | S35 | Seia | PWD-symptomatic | *Ophiostoma ips* | OK559540 | 100.00 | 0.00 |  |
| S35.2 | S35 | Seia | PWD-symptomatic | *Ophiostoma ips* | OK559540 | 100.00 | 0.00 |  |
| S35.3 | S35 | Seia | PWD-symptomatic | *Ophiostoma ips* | OK559542 | 100.00 | 0.00 |  |
| S35.4 | S35 | Seia | PWD-symptomatic | *Ophiostoma ips* | OK559539 | 99.85 | 0.00 |  |
| S35.5 | S35 | Seia | PWD-symptomatic | *Ophiostoma ips* | OK559539 | 100.00 | 0.00 |  |
| S35.7 | S35 | Seia | PWD-symptomatic | *Ophiostoma ips* | MH324814 | 100.00 | 0.00 |  |
| S35.8 | S35 | Seia | PWD-symptomatic | *Ophiostoma ips* | OK559539 | 99.84 | 0.00 |  |
| S36.1 | S36 | Seia | PWD-symptomatic | *Ophiostoma ips* | OK559539 | 100.00 | 0.00 |  |
| S36.3 | S36 | Seia | PWD-symptomatic | *Ophiostoma ips* | OK559539 | 100.00 | 0.00 |  |
| S36.7 | S36 | Seia | PWD-symptomatic | *Ophiostoma ips* | GU170426 | 100.00 | 0.00 |  |
| S36.8 | S36 | Seia | PWD-symptomatic | *Ophiostoma ips* | GU170426 | 100.00 | 0.00 |  |
| S36.9 | S36 | Seia | PWD-symptomatic | *Ophiostoma ips* | AY172021 | 99.84 | 0.00 |  |
| S36.10 | S36 | Seia | PWD-symptomatic | *Ophiostoma ips* | AY172021 | 100.00 | 0.00 |  |
| S38.1 | S38 | Seia | PWD-symptomatic | *Ophiostoma ips* | AY172021 | 100.00 | 0.00 |  |
| S38.3 | S38 | Seia | PWD-symptomatic | *Grosmannia aurea* | OK559543 | 100.00 | 0.00 |  |
| S38.5 | S38 | Seia | PWD-symptomatic | *Ophiostoma ips* | OK559539 | 100.00 | 0.00 |  |
| S38.9 | S38 | Seia | PWD-symptomatic | *Grosmannia aurea T* | NR_145307 | 100.00 | 0.00 |  |
| S38.16 | S38 | Seia | PWD-symptomatic | *Ophiostoma ips* | OK559541 | 100.00 | 0.00 |  |
| S39.1 | S39 | Seia | PWD-symptomatic | *Ophiostoma ips* | OK559540 | 100.00 | 0.00 |  |
| S39.2 | S39 | Seia | PWD-symptomatic | *Ophiostoma ips* | OK559540 | 100.00 | 0.00 |  |
| S39.3 | S39 | Seia | PWD-symptomatic | *Ophiostoma ips* | OK559539 | 99.84 | 0.00 |  |
| S39.5 | S39 | Seia | PWD-symptomatic | *Ophiostoma ips* | OK559539 | 100.00 | 0.00 |  |
| S39.6 | S39 | Seia | PWD-symptomatic | *Ophiostoma ips* | MH324814 | 99.83 | 0.00 |  |
| S39.8 | S39 | Seia | PWD-symptomatic | *Ophiostoma ips* | AY172021 | 100.00 | 0.00 |  |
| S39.9 | S39 | Seia | PWD-symptomatic | *Ophiostoma ips* | OK559539 | 100.00 | 0.00 |  |
| S40.1a | S40 | Seia | PWD-symptomatic | *Ophiostoma ips* | AY172021 | 100.00 | 0.00 |  |
| S40.2a | S40 | Seia | PWD-symptomatic | *Ophiostoma ips* | GU170426 | 100.00 | 0.00 |  |
| S40.5a | S40 | Seia | PWD-symptomatic | *Ophiostoma ips* | GU170426 | 99.83 | 0.00 |  |
| S40.4b | S40 | Seia | PWD-symptomatic | *Leptographium sp.* | OK559544 | 100.00 | 0.00 |  |
| S40.5b | S40 | Seia | PWD-symptomatic | *Leptographium sp.* | OK559544 | 100.00 | 0.00 |  |
| S40.6 | S40 | Seia | PWD-symptomatic | *Uncultured Ophiostoma sp.* | KU663983 | 100.00 | 0.00 |  |
| S40.7 | S40 | Seia | PWD-symptomatic | *Ophiostoma ips* | OK559542 | 100.00 | 0.00 |  |
| S40.10 | S40 | Seia | PWD-symptomatic | *Ophiostoma ips* | OK559542 | 100.00 | 0.00 |  |

**Supplementary Table 2** (continued) – Best BLAST hit of each fungal isolate based upon TUB partial sequence retrieved from the NCBI GenBank database.

| **Isolate ID** | **Tree ID** | **Origin** | ***Pinus pinaster* symptom** | **BLAST nucleotide** | | | |
| --- | --- | --- | --- | --- | --- | --- | --- |
|  |  |  |  | **Description** | **Accession** | **Identity (%)** | **E-value** |
| L2.1 | L2 | Companhia das Lezirias | PWD-symptomatic | *Graphilbum acuminatum* | MN548939.1 | 93.66 | 3,00E-77 |
| L2.2 | L2 | Companhia das Lezirias | PWD-symptomatic | *Graphilbum acuminatum* | MN548939.1 | 93.66 | 3,00E-77 |
| L2.3 | L2 | Companhia das Lezirias | PWD-symptomatic | *Graphilbum acuminatum* | MN548939.1 | 93.66 | 3,00E-77 |
| L2.5 | L2 | Companhia das Lezirias | PWD-symptomatic | *Graphilbum acuminatum* | MN548939.1 | 93.66 | 3,00E-77 |
| L2.6 | L2 | Companhia das Lezirias | PWD-symptomatic | *Leptographium sosnaicola* | MW540773.1 | 98.67 | 0.0 |
| L3.1 | L3 | Companhia das Lezirias | PWD-symptomatic | *Ophiostoma ips* | MH324807.1 | 99.67 | 2,00E-151 |
| L3.2 | L3 | Companhia das Lezirias | PWD-symptomatic | *Ophiostoma ips* | MH324807.1 | 99.35 | 3,00E+155 |
| L3.3 | L3 | Companhia das Lezirias | PWD-symptomatic | *Ophiostoma ips* | MH324807.1 | 99.67 | 6,00E-152 |
| L4.1 | L4 | Companhia das Lezirias | PWD-symptomatic | *Ophiostoma ips* | MH324807.1 | 99.01 | 2,00E-151 |
| L5.2 | L5 | Companhia das Lezirias | PWD-symptomatic | *Ophiostoma ips* | MH324807.1 | 100 | 1,00E-147 |
| L5.3 | L5 | Companhia das Lezirias | PWD-symptomatic | *Sporothrix pseudoabietina* | MH683598 | 100 | 3.24E-144 |
| T1.2 | T1 | Tróia | PWD-symptomatic | *Ophiostoma ips* | MH324807.1 | 100 | 6.73E-148 |
| T1.4 | T1 | Tróia | PWD-symptomatic | *Ophiostoma ips* | MH324807.1 | 100 | 2.63E-145 |
| T1.5 | T1 | Tróia | PWD-symptomatic | *Ophiostoma ips* | MH324807.1 | 100 | 9.14E-145 |
| T1.9 | T1 | Tróia | PWD-symptomatic | *Ophiostoma ips* | MH324807.1 | 100 | 3.43E-145 |
| T1.10 | T1 | Tróia | PWD-symptomatic | *Ophiostoma ips* | MH324807.1 | 100 | 2.63E-145 |
| T1.17 | T1 | Tróia | PWD-symptomatic | *Ophiostoma ips* | MH324807.1 | 100 | 6.26E-147 |
| T2.3 | T1 | Tróia | PWD-symptomatic | *Ophiostoma ips* | MH324807.1 | 100 | 2.63E-145 |
| T2.4 | T2 | Tróia | PWD-symptomatic | *Ophiostoma ips* | MH324807.1 | 100 | 2.93E-151 |
| T2.5 | T2 | Tróia | PWD-symptomatic | *Ophiostoma ips* | MH324807.1 | 100 | 1.80E-147 |
| T2.13 | T3 | Tróia | PWD-symptomatic | Leptographium pini-densiflorae | KR012447 | 97.00 | 3.36E-177 |
| T2.16 | T4 | Tróia | PWD-symptomatic | Leptographium pini-densiflorae | KR012447 | 97.26 | 1.71E-174 |
| T3.1 | T3 | Tróia | PWD-symptomatic | *Leptographium wingfieldii* | GU370186 | 100.00 | 0.00 |
| T3.2 | T3 | Tróia | PWD-symptomatic | *Leptographium wingfieldii* | GU370186 | 99.69 | 0.00 |
| T3.3 | T3 | Tróia | PWD-symptomatic | *Leptographium wingfieldii* | GU370186 | 100.00 | 0.00 |
| T3.5 | T3 | Tróia | PWD-symptomatic | *Leptographium wingfieldii* | GU370186 | 100.00 | 0.00 |
| T3.7 | T3 | Tróia | PWD-symptomatic | *Leptographium wingfieldii* | GU370186 | 99.69 | 0.00 |
| T3.8 | T3 | Tróia | PWD-symptomatic | *Leptographium wingfieldii* | GU370186 | 100.00 | 0.00 |
| T3.9 | T3 | Tróia | PWD-symptomatic | *Leptographium wingfieldii* | GU370186 | 99.69 | 0.00 |
| T7.1 | T7 | Tróia | PWD-symptomatic | *Leptographium pini-densiflorae* | KR012447 | 4.03E-176 | 97.26 |
| T7.2 | T7 | Tróia | PWD-symptomatic | *Ophiostoma ips* | MH324807.1 | 98.95 | 8.50E-139 |
| T7.3 | T7 | Tróia | PWD-symptomatic | *Ophiostoma ips* | MH324807.1 | 100.00 | 2.63E-145 |
| T7.4 | T7 | Tróia | PWD-symptomatic | *Ophiostoma ips* | MH324807.1 | 100.00 | 8.42E-152 |
| T7.5 | T7 | Tróia | PWD-symptomatic | *Ophiostoma ips* | MH324807.1 | 100.00 | 8.42E-152 |
| T7.6 | T7 | Tróia | PWD-symptomatic | *Ophiostoma ips* | MH324807.1 | 100.00 | 2.63E-145 |
| T7.7 | T7 | Tróia | PWD-symptomatic | *Ophiostoma ips* | MH324807.1 | 99.66 | 2.69E-145 |
| T7.8 | T7 | Tróia | PWD-symptomatic | *Ophiostoma ips* | MH324807.1 | 100.00 | 8.42E-152 |
| T7.9 | T7 | Tróia | PWD-symptomatic | *Ophiostoma ips* | MH324807.1 | 100.00 | 3.92E-143 |
| S13.1 | S13 | Seia | PWD-symptomatic | *Ophiostoma ips* | MH324807 | 100.00 | 2.94E-151 |
| S19.2 | S19 | Seia | PWD-symptomatic | *Ophiostoma ips* | MH324807 | 99.66 | 3.24E-144 |
| S19.16 | S19 | Seia | PWD-symptomatic | *Ophiostoma ips* | MH324807 | 100.00 | 1.24E-149 |
| S19.17 | S19 | Seia | PWD-symptomatic | *Ophiostoma ips* | MH324807 | 100.00 | 2.64E-145 |
| S19.19 | S19 | Seia | PWD-symptomatic | *Ophiostoma ips* | MH324807 | 100.00 | 2.36E-139 |
| S32.10 | S32 | Seia | PWD-symptomatic | *Ophiostoma ips* | MH324807 | 100.00 | 2.18E-146 |
| S35.1 | S35 | Seia | PWD-symptomatic | *Ophiostoma ips* | MH324807 | 100.00 | 3.24E-144 |
| S35.2 | S35 | Seia | PWD-symptomatic | *Ophiostoma ips* | MH324807 | 100.00 | 1.13E-143 |
| S35.3 | S35 | Seia | PWD-symptomatic | *Ophiostoma ips* | MH324807 | 100.00 | 2.36E-139 |
| S35.4 | S35 | Seia | PWD-symptomatic | *Ophiostoma ips* | MH324807 | 100.00 | 3.24E-144 |
| S35.5 | S35 | Seia | PWD-symptomatic | *Ophiostoma ips* | MH324807 | 100.00 | 5.63E-141 |
| S35.7 | S35 | Seia | PWD-symptomatic | *Ophiostoma ips* | MH324807 | 100.00 | 3.26E-144 |
| S35.8 | S35 | Seia | PWD-symptomatic | *Ophiostoma ips* | MH324807 | 100.00 | 4.11E-169 |
| S36.1 | 36 | Seia | PWD-symptomatic | *Ophiostoma ips* | MH324807 | 100.00 | 8.22E-139 |
| S36.3 | 36 | Seia | PWD-symptomatic | *Ophiostoma ips* | MH324807 | 100.00 | 4.41E-149 |
| S36.5 | 36 | Seia | PWD-symptomatic | *Ophiostoma ips* | MH324807 | 100.00 | 5.31E-148 |
| S36.7 | 36 | Seia | PWD-symptomatic | *Ophiostoma ips* | MH324807 | 100.00 | 4.39E-149 |
| S36.8 | 36 | Seia | PWD-symptomatic | *Ophiostoma ips* | MH324807 | 100.00 | 5.81E-141 |
| S36.9 | 36 | Seia | PWD-symptomatic | *Ophiostoma ips* | MH324807 | 99.66 | 6.52E-147 |
| S36.10 | 36 | Seia | PWD-symptomatic | *Ophiostoma ips* | MH324807 | 100.00 | 4.30E-149 |
| S38.1 | S38 | Seia | PWD-symptomatic | *Ophiostoma ips* | MH324807 | 100.00 | 1.34E-142 |
| S38.3 | S38 | Seia | PWD-symptomatic | *Leptographium terebrantis* | GU370186 | 100.00 | 0.00 |
| S38.5 | S38 | Seia | PWD-symptomatic | *Ophiostoma ips* | MH324807 | 100.00 | 5.35E-148 |
| S38.6 | S38 | Seia | PWD-symptomatic | *Leptographium terebrantis* | GU370186 | 100.00 | 0.00 |
| S38.9 | S38 | Seia | PWD-symptomatic | *Leptographium terebrantis* | GU370186 | 100.00 | 0.00 |
| S38.15 | S38 | Seia | PWD-symptomatic | *Leptographium terebrantis* | GU370186 | 100.00 | 0.00 |
| S38.16 | S38 | Seia | PWD-symptomatic | *Ophiostoma ips* | MH324807 | 100.00 | 7.59E-146 |
| S39.2 | S39 | Seia | PWD-symptomatic | *Ophiostoma ips* | MH324807 | 100.00 | 7.59E-146 |
| S39.3 | S39 | Seia | PWD-symptomatic | *Ophiostoma ips* | MH324807 | 100.00 | 8.45E-152 |
| S39.4 | S39 | Seia | PWD-symptomatic | *Ophiostoma ips* | MH324807 | 100.00 | 8.45E-152 |
| S39.5 | S39 | Seia | PWD-symptomatic | *Ophiostoma ips* | MH324807 | 100.00 | 2.94E-151 |
| S39.6 | S39 | Seia | PWD-symptomatic | *Ophiostoma ips* | MH324807 | 100.00 | 1.66E-154 |
| S39.8 | S39 | Seia | PWD-symptomatic | *Ophiostoma ips* | MH324807 | 100.00 | 6.99E-153 |
| S39.9 | S39 | Seia | PWD-symptomatic | *Ophiostoma ips* | MH324807 | 100.00 | 2.02E-153 |
| S40.1a | S40 | Seia | PWD-symptomatic | *Ophiostoma ips* | MH324807 | 100.00 | 7.01E-153 |
| S40.1b | S40 | Seia | PWD-symptomatic | *Leptographium terebrantis* | GU370186 | 100.00 | 0.00 |
| S40.2a | S40 | Seia | PWD-symptomatic | *Ophiostoma ips* | MH324807 | 100.00 | 1.25E-149 |
| S40.4a | S40 | Seia | PWD-symptomatic | *Ophiostoma ips* | MH324807 | 100.00 | 2.43E-152 |
| S40.5a | S40 | Seia | PWD-symptomatic | *Ophiostoma ips* | MH324807 | 100.00 | 8.35E-139 |
| S40.4b | S40 | Seia | PWD-symptomatic | *Leptographium terebrantis* | GU370186 | 100.00 | 0.00 |
| S40.5B | S40 | Seia | PWD-symptomatic | *Leptographium terebrantis* | GU370186 | 100.00 | 0.00 |
| S40.6 | S40 | Seia | PWD-symptomatic | *Ophiostoma ips* | MH324807 | 100.00 | 7.09E-153 |
| S40.7 | S40 | Seia | PWD-symptomatic | *Ophiostoma ips* | MH324807 | 100.00 | 6.28E-147 |
| S40.10 | S40 | Seia | PWD-symptomatic | *Ophiostoma ips* | MH324807 | 100.00 | 3.55E-150 |
| S40.11 | S40 | Seia | PWD-symptomatic | *Leptographium terebrantis* | GU370186 | 100.00 | 0.00 |

**Supplementary Table 2** (continued) – Best BLAST hit of each fungal isolate based upon TEF partial sequence retrieved from the NCBI GenBank database.

| **Isolate ID** | **Tree ID** | **Origin** | ***Pinus pinaster* symptoms** | **NCBI Nucleotide** | | | |
| --- | --- | --- | --- | --- | --- | --- | --- |
|  |  |  |  | ***Description*** | **Accession** | **Identity (%)** | **E-value** |
| L2.1 | L2 | Companhia das Lezirias | PWD-symptomatic | *Graphilbum acuminatum* | MN548952 | 95.98 | 1,00E-71 |
| L2.2 | L2 | Companhia das Lezirias | PWD-symptomatic | *Graphilbum acuminatum* | MN548952 | 96.55 | 3,00E-73 |
| L2.3 | L2 | Companhia das Lezirias | PWD-symptomatic | *Graphilbum acuminatum* | MN548952 | 96.55 | 3,00E-73 |
| L2.5 | L2 | Companhia das Lezirias | PWD-symptomatic | *Graphilbum acuminatum* | MN548952 | 95.03 | 1,00E-71 |
| L2.6 | L2 | Companhia das Lezirias | PWD-symptomatic | *Leptographium sosnaicola* | MW540787 | 99.12 | 0.0 |
| L3.1 | L3 | Companhia das Lezirias | PWD-symptomatic | *Ophiostoma ips* | MG702098 | 99.82 | 0.0 |
| L3.2 | L3 | Companhia das Lezirias | PWD-symptomatic | *Ophiostoma ips* | MG702098 | 100 | 0.0 |
| L3.3 | L3 | Companhia das Lezirias | PWD-symptomatic | *Ophiostoma ips* | MG702098 | 99.82 | 0.0 |
| L4.1 | L4 | Companhia das Lezirias | PWD-symptomatic | *Ophiostoma ips* | MG702098 | 100 | 0.0 |
| L4.2 | L4 | Companhia das Lezirias | PWD-symptomatic | *Graphilbum acuminatum* | MN548952 | 97 | 8,00E-74 |
| L5.1 | L5 | Companhia das Lezirias | PWD-symptomatic | *Graphilbum acuminatum* | MN548952 | 87 | 2,00E-74 |
| L5.2 | L5 | Companhia das Lezirias | PWD-symptomatic | *Ophiostoma ips* | MG702098 | 99.82 | 0.0 |
| L5.3 | L5 | Companhia das Lezirias | PWD-symptomatic | *Sporothrix sp.* | KY568667 | 99.39 | 0.0 |
| T1.2 | T1 | Tróia | PWD-symptomatic | *Ophiostoma ips* | MG702098 | 100.00 | 0.00 |
| T1.3 | T1 | Tróia | PWD-symptomatic | *Ophiostoma ips* | MG702098 | 100.00 | 0.00 |
| T1.4 | T1 | Tróia | PWD-symptomatic | *Ophiostoma ips* | MG702098 | 100.00 | 0.00 |
| T1.5 | T1 | Tróia | PWD-symptomatic | *Ophiostoma ips* | MG702098 | 100.00 | 0.00 |
| T1.9 | T1 | Tróia | PWD-symptomatic | *Ophiostoma ips* | MG702098 | 100.00 | 0.00 |
| T1.10 | T1 | Tróia | PWD-symptomatic | *Ophiostoma ips* | MG702098 | 100.00 | 0.00 |
| T1.17 | T1 | Tróia | PWD-symptomatic | *Ophiostoma ips* | MG702098 | 99.80 | 0.00 |
| T2.3 | T2 | Tróia | PWD-symptomatic | *Ophiostoma ips* | MG702098 | 100.00 | 0.00 |
| T2.4 | T2 | Tróia | PWD-symptomatic | *Ophiostoma ips* | MG702098 | 100.00 | 0.00 |
| T2.5 | T2 | Tróia | PWD-symptomatic | *Ophiostoma ips* | MG702098 | 99.80 | 0.00 |
| T2.18 | T2 | Tróia | PWD-symptomatic | *Ophiostoma ips* | MG702098 | 100.00 | 0.00 |
| T3.1 | T3 | Tróia | PWD-symptomatic | *Leptographium terebrantis* | GU370251 | 99.31 | 0.00 |
| T3.2 | T3 | Tróia | PWD-symptomatic | *Leptographium terebrantis* | GU370251 | 98.85 | 0.00 |
| T3.3 | T3 | Tróia | PWD-symptomatic | *Leptographium terebrantis* | GU370251 | 99.31 | 0.00 |
| T3.5 | T3 | Tróia | PWD-symptomatic | *Leptographium terebrantis* | GU370251 | 99.08 | 0.00 |
| T3.6 | T3 | Tróia | PWD-symptomatic | *Leptographium terebrantis* | GU370251 | 99.31 | 0.00 |
| T3.8 | T3 | Tróia | PWD-symptomatic | *Leptographium terebrantis* | GU370251 | 99.07 | 0.00 |
| T3.9 | T3 | Tróia | PWD-symptomatic | *Leptographium terebrantis* | GU370251 | 99.30 | 0.00 |
| T7.2 | T7 | Tróia | PWD-symptomatic | *Ophiostoma ips* | MG702098 | 100.00 | 0.00 |
| T7.3 | T7 | Tróia | PWD-symptomatic | *Ophiostoma ips* | MG702098 | 100.00 | 0.00 |
| T7.4 | T7 | Tróia | PWD-symptomatic | *Ophiostoma ips* | MG702098 | 100.00 | 0.00 |
| T7.5 | T7 | Tróia | PWD-symptomatic | *Ophiostoma ips* | MG702098 | 100.00 | 0.00 |
| T7.7 | T7 | Tróia | PWD-symptomatic | *Ophiostoma ips* | MG702098 | 100.00 | 0.00 |
| T7.6 | T7 | Tróia | PWD-symptomatic | *Ophiostoma ips* | MG702098 | 100.00 | 0.00 |
| T7.8 | T7 | Tróia | PWD-symptomatic | *Ophiostoma ips* | MG702098 | 100.00 | 0.00 |
| T7.9 | T7 | Tróia | PWD-symptomatic | *Ophiostoma ips* | MG702098 | 100.00 | 0.00 |
| S13.1 | S13 | Seia | Non-symptomatic | *Ophiostoma ips* | MG702098 | 100.00 | 0.00 |
| S19.2 | S19 | Seia | Non-symptomatic | *Ophiostoma ips* | MG702098 | 99.81 | 0.00 |
| S19.15 | S19 | Seia | Non-symptomatic | *Ophiostoma ips* | MG702098 | 99.61 | 0.00 |
| S19.16 | S19 | Seia | Non-symptomatic | *Ophiostoma ips* | KU319147 | 99.81 | 0.00 |
| S19.17 | S19 | Seia | Non-symptomatic | *Ophiostoma ips* | MH195229 | 99.81 | 0.00 |
| S19.19 | S19 | Seia | Non-symptomatic | *Ophiostoma ips* | MG702098 | 100.00 | 0.00 |
| S32.10 | S32 | Seia | Non-symptomatic | *Ophiostoma ips* | KU319150 | 100.00 | 0.00 |
| S35.1 | S35 | Seia | PWD-symptomatic | *Ophiostoma ips* | MG702098 | 100.00 | 0.00 |
| S35.2 | S35 | Seia | PWD-symptomatic | *Ophiostoma ips* | MG702098 | 99.80 | 0.00 |
| S35.3 | S35 | Seia | PWD-symptomatic | *Ophiostoma ips* | MG702098 | 100.00 | 0.00 |
| S35.4 | S35 | Seia | PWD-symptomatic | *Ophiostoma ips* | MG702098 | 99.82 | 0.00 |
| S35.5 | S35 | Seia | PWD-symptomatic | *Ophiostoma ips* | KU319150 | 100.00 | 0.00 |
| S35.7 | S35 | Seia | PWD-symptomatic | *Ophiostoma ips* | MG702098 | 100.00 | 0.00 |
| S36.1 | S36 | Seia | PWD-symptomatic | *Ophiostoma ips* | MG702098 | 100.00 | 0.00 |
| S36.3 | S36 | Seia | PWD-symptomatic | *Ophiostoma ips* | KU319150 | 100.00 | 0.00 |
| S36.5 | S36 | Seia | PWD-symptomatic | *Ophiostoma ips* | MG702098 | 99.62 | 0.00 |
| S36.7 | S36 | Seia | PWD-symptomatic | *Ophiostoma ips* | MG702098 | 100.00 | 0.00 |
| S36.8 | S36 | Seia | PWD-symptomatic | *Ophiostoma ips* | MG702098 | 100.00 | 0.00 |
| S36.9 | S36 | Seia | PWD-symptomatic | *Ophiostoma ips* | MG702098 | 100.00 | 0.00 |
| S36.10 | S36 | Seia | PWD-symptomatic | *Ophiostoma ips* | MG702098 | 100.00 | 0.00 |
| S38.1 | S38 | Seia | PWD-symptomatic | *Ophiostoma ips* | MG702098 | 100.00 | 0.00 |
| S38.3 | S38 | Seia | PWD-symptomatic | *Leptographium terebrantis* | GU370251 | 99.77 | 0.00 |
| S38.5 | S38 | Seia | PWD-symptomatic | *Ophiostoma ips* | MG702098 | 100.00 | 0.00 |
| S38.6 | S38 | Seia | PWD-symptomatic | *Leptographium terebrantis* | GU370251 | 99.54 | 0.00 |
| S38.7 | S38 | Seia | PWD-symptomatic | *Graphilbum ipis-grandicollis* | MW066405 | 97.62 | 5.99E-107 |
| S38.9 | S38 | Seia | PWD-symptomatic | *Leptographium terebrantis* | GU370251 | 99.78 | 0.00 |
| S38.16 | S38 | Seia | PWD-symptomatic | *Ophiostoma ips* | MG702098 | 100.00 | 0.00 |
| S38.15 | S38 | Seia | PWD-symptomatic | *Leptographium terebrantis* | GU370251 | 99.77 | 0.00 |
| S38.16 | S38 | Seia | PWD-symptomatic | *Ophiostoma ips* | MG702098 | 100.00 | 0.00 |
| S39.2 | S39 | Seia | PWD-symptomatic | *Ophiostoma ips* | MG702098 | 99.80 | 0.00 |
| S39.3 | S39 | Seia | PWD-symptomatic | *Ophiostoma ips* | MH195229 | 99.82 | 0.00 |
| S39.4 | S39 | Seia | PWD-symptomatic | *Ophiostoma ips* | MG702098 | 100.00 | 0.00 |
| S39.5 | S39 | Seia | PWD-symptomatic | *Ophiostoma ips* | KU319148 | 99.62 | 0.00 |
| S39.6 | S39 | Seia | PWD-symptomatic | *Ophiostoma ips* | KU319147 | 99.81 | 0.00 |
| S39.8 | S39 | Seia | PWD-symptomatic | *Ophiostoma ips* | MG702098 | 99.80 | 0.00 |
| S39.9 | S39 | Seia | PWD-symptomatic | *Ophiostoma ips* | MG702098 | 99.61 | 0.00 |
| S40.1a | S40 | Seia | PWD-symptomatic | *Ophiostoma ips* | KU319150 | 99.79 | 0.00 |
| S40.1b | S40 | Seia | PWD-symptomatic | *Leptographium terebrantis* | GU370225 | 99.25 | 0.00 |
| S40.2a | S40 | Seia | PWD-symptomatic | *Ophiostoma ips* | MG702098 | 100.00 | 0.00 |
| S40.4a | S40 | Seia | PWD-symptomatic | *Ophiostoma ips* | MG702098 | 100.00 | 0.00 |
| S40.4b | S40 | Seia | PWD-symptomatic | *Leptographium terebrantis* | GU370251 | 98.86 | 0.00 |
| S40.5a | S40 | Seia | PWD-symptomatic | *Ophiostoma ips* | KU319150 | 100.00 | 0.00 |
| S40.5b | S40 | Seia | PWD-symptomatic | *Leptographium terebrantis* | GU370251 | 98.86 | 0.00 |
| S40.6 | S40 | Seia | PWD-symptomatic | *Ophiostoma ips* | MG702098 | 100.00 | 0.00 |
| S40.7 | S40 | Seia | PWD-symptomatic | *Ophiostoma ips* | MG702098 | 100.00 | 0.00 |
| S40.10 | S40 | Seia | PWD-symptomatic | *Ophiostoma ips* | MG702098 | 100.00 | 0.00 |
| S40.11 | S40 | Seia | PWD-symptomatic | *Leptographium terebrantis* | GU370251 | 99.09 | 0.00 |

**Supplementary Table 2** (continued) – Best BLAST hit of each fungal isolate based upon CAL partial sequence retrieved from the NCBI GenBank database.

| **Isolate ID** | **Tree ID** | **Origin** | ***Pinus pinaster* symptoms** | **NCBI Nucleotide** | | | |
| --- | --- | --- | --- | --- | --- | --- | --- |
|  |  |  |  | ***Description*** | **Accession** | **Identity (%)** | **E-value** |
| L2.1 | L2 | Companhia das Lezirias | PWD-symptomatic | *Graphilbum sp.* | MN548987.1 | 94.59 | 2,00E-139 |
| L2.2 | L2 | Companhia das Lezirias | PWD-symptomatic | *Graphilbum sp.* | MN548987.1 | 94.58 | 8,00E-139 |
| L2.3 | L2 | Companhia das Lezirias | PWD-symptomatic | *Graphilbum sp.* | MN548987.1 | 94.56 | 3,00E-138 |
| L2.6 | L2 | Companhia das Lezirias | PWD-symptomatic | *Leptographium sosnaicola* | MT210412.1 | 97.74 | 0.0 |
| L3.1 | L3 | Companhia das Lezirias | PWD-symptomatic | *Ophiostoma ips* | MW075141.1 | 99.14 | 0.0 |
| L3.2 | L3 | Companhia das Lezirias | PWD-symptomatic | *Ophiostoma ips* | MW075141.1 | 99.77 | 0.0 |
| L3.3 | L3 | Companhia das Lezirias | PWD-symptomatic | *Ophiostoma ips* | MW075141.1 | 99.78 | 0.0 |
| L5.3 | L5 | Companhia das Lezirias | PWD-symptomatic | *Ophiostoma ips* | MW075141.1 | 99.57 | 0.0 |
| T1.4 | T1 | Tróia | PWD-symptomatic | *Ophiostoma ips* | MW075141 | 99.21 | 0.00 |
| T2.3 | T2 | Tróia | PWD-symptomatic | *Ophiostoma ips* | MW075141 | 99.31 | 0.00 |
| T2.13 | T2 | Tróia | PWD-symptomatic | *Leptographium sosnaicola* | MT210412 | 100.00 | 0.00 |
| T2.16 | T2 | Tróia | PWD-symptomatic | *Leptographium gracile* | MG205782 | 96.50 | 0.00 |
| T3.1 | T3 | Tróia | PWD-symptomatic | *Grosmannia clavigera* | KP171178 | 100.00 | 0.00 |
| T3.2 | T3 | Tróia | PWD-symptomatic | *Grosmannia clavigera* | KP171178 | 100.00 | 0.00 |
| T3.3 | T3 | Tróia | PWD-symptomatic | *Grosmannia clavigera* | KP171178 | 100.00 | 0.00 |
| T3.5 | T3 | Tróia | PWD-symptomatic | *Grosmannia clavigera* | KP171178 | 100.00 | 0.00 |
| T3.6 | T3 | Tróia | PWD-symptomatic | *Grosmannia clavigera* | KP171178 | 100.00 | 0.00 |
| T3.7 | T3 | Tróia | PWD-symptomatic | *Grosmannia clavigera* | KP171178 | 100.00 | 0.00 |
| T3.9 | T3 | Tróia | PWD-symptomatic | *Grosmannia clavigera* | KP171178 | 99.21 | 0.00 |
| T7.1 | T7 | Tróia | PWD-symptomatic | *Leptographium gracile* | MG205782 | 98.49 | 5.65E-170 |
| T7.2 | T7 | Tróia | PWD-symptomatic | *Ophiostoma ips* | MW075141 | 99.38 | 2.27E-159 |
| T7.4 | T7 | Tróia | PWD-symptomatic | *Ophiostoma ips* | MW075141 | 99.75 | 0.00 |
| T7.8 | T7 | Tróia | PWD-symptomatic | *Ophiostoma ips* | MW075141 | 99.31 | 0.00 |
| S13.1 | S13 | Seia | Non-symptomatic | *Ophiostoma ips* | MW075141 | 99.70 | 0.00 |
| S19.2 | S19 | Seia | Non-symptomatic | *Ophiostoma ips* | MW075141 | 99.39 | 0.00 |
| S19.15 | S19 | Seia | Non-symptomatic | *Ophiostoma ips* | MW075141 | 99.54 | 0.00 |
| S19.16 | S19 | Seia | Non-symptomatic | *Ophiostoma ips* | MW075141 | 99.54 | 0.00 |
| S19.17 | S19 | Seia | Non-symptomatic | *Ophiostoma ips* | MW075141 | 99.38 | 0.00 |
| S19.19 | S19 | Seia | Non-symptomatic | *Ophiostoma ips* | MW075141 | 99.24 | 0.00 |
| S32.10 | S32 | Seia | Non-symptomatic | *Ophiostoma ips* | MW075141 | 99.23 | 0.00 |
| S35.1 | S35 | Seia | PWD-symptomatic | *Ophiostoma ips* | MW075141 | 99.39 | 0.00 |
| S35.2 | S35 | Seia | PWD-symptomatic | *Ophiostoma ips* | MW075141 | 99.69 | 0.00 |
| S35.3 | S35 | Seia | PWD-symptomatic | *Ophiostoma ips* | MW075141 | 99.69 | 0.00 |
| S35.7 | S35 | Seia | PWD-symptomatic | *Ophiostoma ips* | MW075141 | 99.69 | 0.00 |
| S35.8 | S35 | Seia | PWD-symptomatic | *Ophiostoma ips* | MW075141 | 99.39 | 0.00 |
| S36.3 | S36 | Seia | PWD-symptomatic | *Ophiostoma ips* | MW075141 | 99.25 | 0.00 |
| S36.5 | S36 | Seia | PWD-symptomatic | *Ophiostoma ips* | MW075141 | 99.39 | 0.00 |
| S36.7 | S36 | Seia | PWD-symptomatic | *Ophiostoma ips* | MW075141 | 99.69 | 0.00 |
| S36.8 | S36 | Seia | PWD-symptomatic | *Ophiostoma ips* | MW075141 | 99.09 | 0.00 |
| S36.9 | S36 | Seia | PWD-symptomatic | *Ophiostoma ips* | MW075141 | 99.56 | 0.00 |
| S36.10 | S36 | Seia | PWD-symptomatic | *Ophiostoma ips* | MW075141 | 99.24 | 0.00 |
| S38.1 | S38 | Seia | PWD-symptomatic | *Ophiostoma ips* | MW075141 | 99.54 | 0.00 |
| S38.3 | S38 | Seia | PWD-symptomatic | *Grosmannia clavigera* | XM_014315032 | 100.00 | 0.00 |
| S38.5 | S38 | Seia | PWD-symptomatic | *Ophiostoma ips* | MW075141 | 99.54 | 0.00 |
| S38.9 | S38 | Seia | PWD-symptomatic | *Grosmannia clavigera* | XM_014315032 | 100.00 | 0.00 |
| S38.16 | S38 | Seia | PWD-symptomatic | *Ophiostoma ips* | MW075141 | 99.85 | 0.00 |
| S38.15 | S38 | Seia | PWD-symptomatic | *Grosmannia radiaticola* | MW075128 | 100.00 | 0.00 |
| S38.16 | S38 | Seia | PWD-symptomatic | *Ophiostoma ips* | MW075141 | 99.85 | 0.00 |
| S39.2 | S39 | Seia | PWD-symptomatic | *Ophiostoma ips* | MW075141 | 99.85 | 0.00 |
| S39.3 | S39 | Seia | PWD-symptomatic | *Ophiostoma ips* | MW075141 | 99.25 | 0.00 |
| S39.4 | S39 | Seia | PWD-symptomatic | *Ophiostoma ips* | MW075141 | 99.54 | 0.00 |
| S39.8 | S39 | Seia | PWD-symptomatic | *Ophiostoma ips* | MW075141 | 99.24 | 0.00 |
| S39.9 | S39 | Seia | PWD-symptomatic | *Ophiostoma ips* | MW075141 | 99.55 | 0.00 |
| S40.1a | S40 | Seia | PWD-symptomatic | *Ophiostoma ips* | MW075141 | 99.40 | 0.00 |
| S40.1b | S40 | Seia | PWD-symptomatic | *Grosmannia radiaticola* | MW075128 | 100.00 | 0.00 |
| S40.4a | S40 | Seia | PWD-symptomatic | *Ophiostoma ips* | MW075141 | 99.25 | 0.00 |
| S40.4b | S40 | Seia | PWD-symptomatic | *Ophiostoma ips* | MW075141 | 99.53 | 0.00 |
| S40.5b | S40 | Seia | PWD-symptomatic | *Ophiostoma ips* | MW075141 | 99.53 | 0.00 |
| S40.6 | S40 | Seia | PWD-symptomatic | *Ophiostoma ips* | MW075141 | 99.55 | 0.00 |
| S40.7 | S40 | Seia | PWD-symptomatic | *Ophiostoma ips* | MW075141 | 99.24 | 0.00 |
| S40.10 | S40 | Seia | PWD-symptomatic | *Ophiostoma ips* | MW075141 | 99.54 | 0.00 |
| S40.11 | S40 | Seia | PWD-symptomatic | *Grosmannia radiaticola* | MW075128 | 100.00 | 0.00 |

**Supplementary Table 3** – Reference sequences per locus (ITS, TUB, TEF and CAL) retrieved from the NCBI database for phylogenetic tree construction.

**SupplementaryTable 3** (continued)– Reference sequences per locus (ITS, TUB, TEF and CAL) retrieved from the NCBI database for phylogenetic tree construction.

**SupplementaryTable 3** (continued )– Reference sequences per locus (ITS, TUB, TEF and CAL) retrieved from the NCBI database for phylogenetic tree construction.

**SupplementaryTable 3** (continued)– Reference sequences per locus (ITS, TUB, TEF and CAL) retrieved from the NCBI database for phylogenetic tree construction.
