# Supplementary material for: Fungal Communities of the Pine Wilt Disease Complex: Studying the Interaction of Ophiostomatales With Bursaphelenchus xylophilus
Source: Front Plant Sci. 2022 Jun 14;13:908308. doi: 10.3389/fpls.2022.908308 (PMC9257700; doi:10.3389/fpls.2022.908308)
Supplement: Supplementary file 2 [file Table_2.DOCX]

Supplementary Material


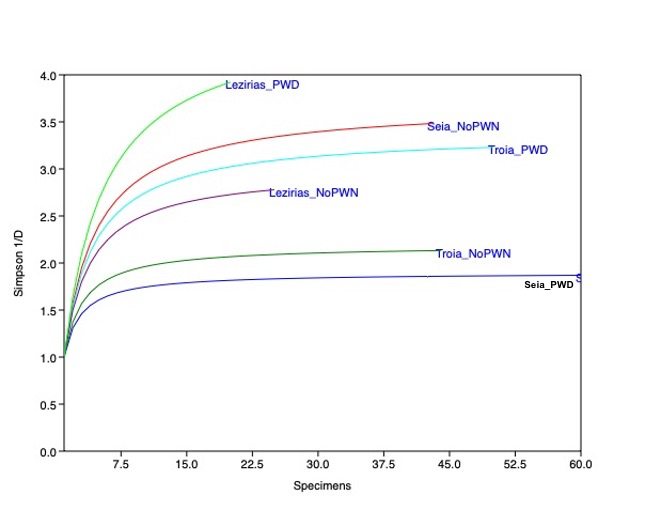


**Supplementary Figure 1** – Rarefaction curves based on Simpson 1/D index for each location site and tree symptomology (No PWN, not infected; PWD, PWN infected).


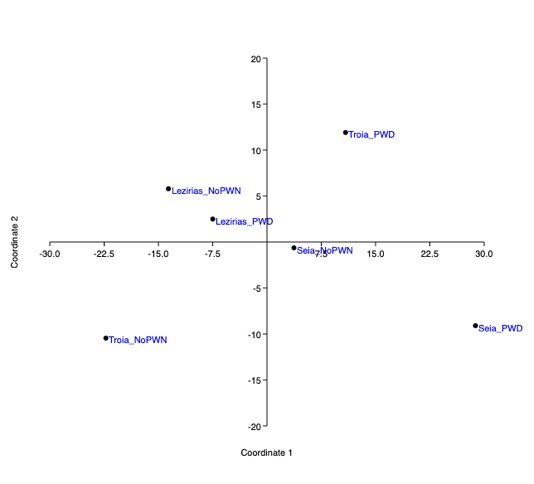


**Supplementary Figure 2** – Principal coordinate analysis for each location site and tree symptomology (No PWN, not infected; PWD, PWN infected).

**Supplementary Figure 3** – Maximum likelihood trees of *Leptographium* sensu lato generated by ITS DNA sequence data. Sequences generated from this study are presented in Table 2. Bold branches indicate bootstrap values ≥ 70%. T=ex-type isolates


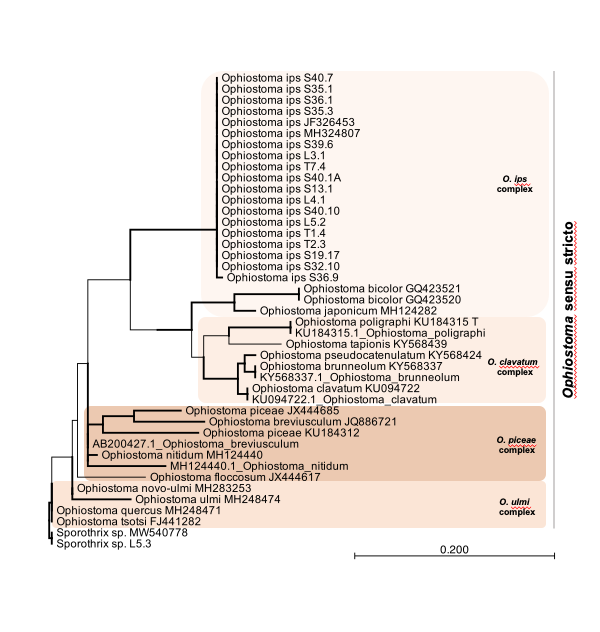


**Supplementary Figure 4** – Maximum likelihood trees of *Ophiostoma sensu stricto* generated by beta-tubulin DNA sequence data. Sequences generated from this study are presented in Table 2. Bold branches indicate bootstrap values ≥ 70%. T=ex-type isolates


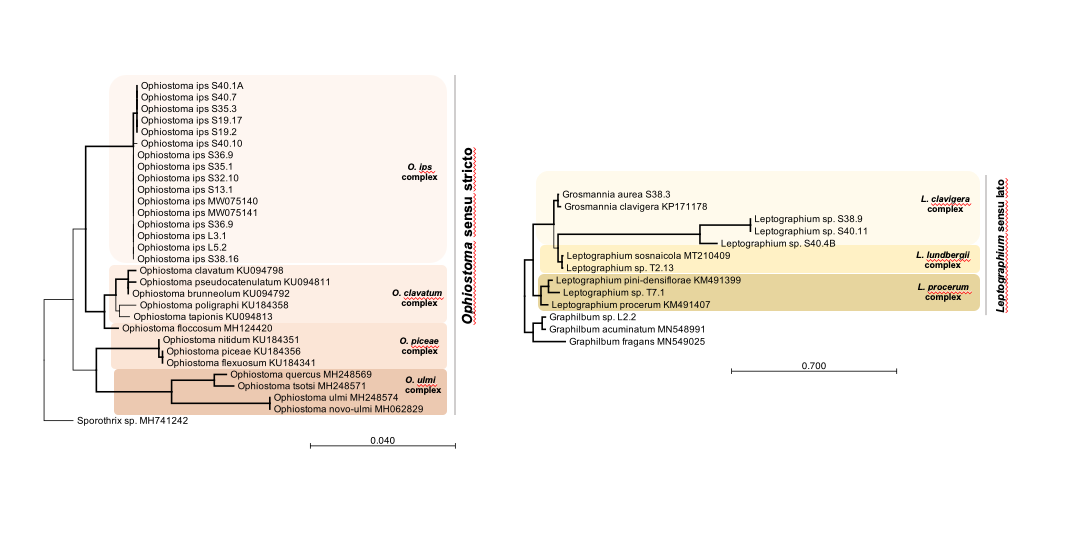


**Supplementary Figure 5** - Maximum likelihood trees of *Ophiostoma sensu stricto* and *Leptographium sensu lato* generated by calmodulin DNA sequence data. Sequences generated from this study are presented in Table 2. Bold branches indicate bootstrap values ≥ 70%. T=ex-type isolates


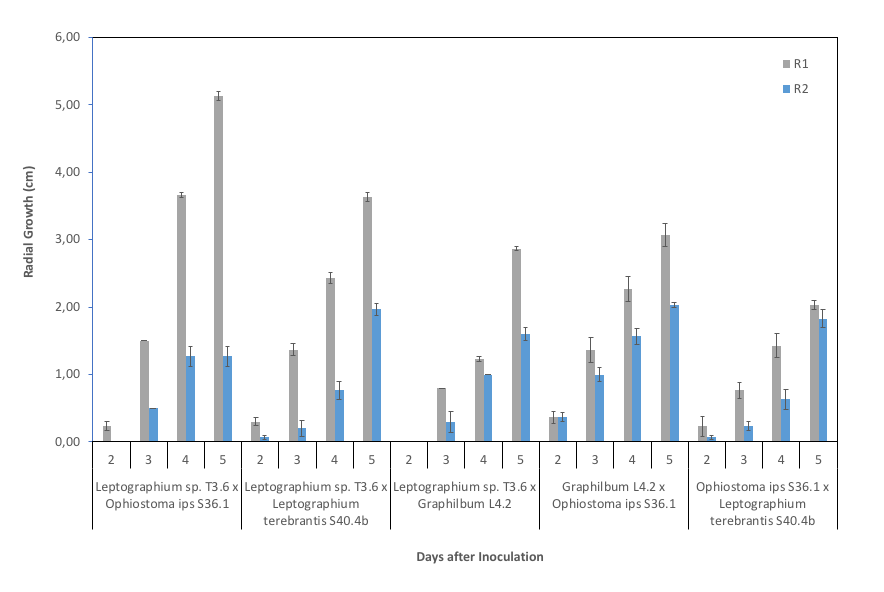


**Supplementary Figure 6** - Radial growth (cm) of the potential antagonist (R1) and tested organism (R2) in Potato dextrose medium during 5 days of incubation at 25ºC.
